# Detection and differentiation of low virulence and virulent *Orthoavulavirus javaense* using a molecular beacon with RT-LAMP

**DOI:** 10.1038/s41598-024-68816-7

**Published:** 2024-08-05

**Authors:** Megan C. Mears, Timothy L. Olivier, Dawn Williams-Coplin, Edna Espinoza, Abhijeet Bakre

**Affiliations:** Exotic and Emerging Avian Viral Disease Research Unit, Southeast Poultry Research Laboratories, US National Poultry Research Center, 934 College Station Road, Athens, GA 30605 USA

**Keywords:** Newcastle disease virus, Loop-mediated isothermal amplification, Diagnostic assay, Virology, Reverse transcription polymerase chain reaction

## Abstract

Newcastle disease (ND), an economically important disease in poultry, is caused by virulent strains of the genetically diverse *Orthoavulavirus javaense* (OAVJ). Laboratories rely on quantitative real-time reverse transcription PCR (qRT-PCR) to detect OAVJ and differentiate between OAVJ pathotypes. This study demonstrates that a fusion cleavage site based molecular beacon with reverse transcription loop mediated isothermal amplification (MB-RT-LAMP) assay can detect and differentiate OAVJ pathotypes in a single assay. Data show that the assay can rapidly identify diverse OAVJ genotypes with sensitivity only one log-fold lower than the current fusion qRT-PCR assay (10^4^ copies), exhibits a high degree of specificity for OAVJ, and the molecular beacon can differentiate mesogenic/velogenic sequences from lentogenic sequences. Further, data show that a two-minute rapid lysis protocol preceding MB-RT-LAMP can detect and differentiate OAVJ RNA from both spiked samples and oropharyngeal swabs without the need for RNA isolation. As the MB-RT-LAMP assay can rapidly detect and discriminate between lentogenic and mesogenic/velogenic sequences of OAVJ within one assay, without the need for RNA isolation, and is adaptable to existing veterinary diagnostic laboratory workflow without additional equipment, this assay could be a rapid primary screening tool before qRT-PCR based validation in resource limited settings.

## Introduction

Newcastle disease (ND) is an economically important disease in poultry^1,2^ and is caused by virulent strains of *Orthoavulavirus javaense* (OAVJ). ND is a listed disease by the World Organization for Animal Health (WOAH; formerly OIE) and can lead to trade embargos, significant economic damage, and loss to the poultry industry^2^. OAVJ virions are pleomorphic enveloped structures, enclosing an approximately 15.2 kb long negative sense single stranded non-segmented RNA (-ssRNA) genome that encodes six genes: nucleocapsid (N), phosphoprotein (P), matrix (M), fusion (F), hemagglutinin-neuraminidase (HN), and polymerase (L)^3^. The F protein is the major determinant of virulence and has been used to classify OAVJ viruses into two major genetic classes, I and II, with class II viruses being primarily involved in ND outbreaks. Class II viruses exhibit a significant amount of genetic diversity and are further sub-classified into 21 genotypes. Within class II, there are three pathotypes of OAVJ: viruses of low virulence are known as lentogens, or lentogenic strains of OAVJ, whereas more virulent OAVJ strains are known as mesogens or velogens. Clinically, virulence is determined by measuring the intra-cerebral pathogenicity index (ICPI)^4^ in 1-day old chickens. Viruses with ICPI scores < 0.7 are lentogenic, while viruses with ICPI scores > 0.7–1.6 are considered mesogenic and those with ICPI > 1.7–2.0 are considered velogenic OAVJ^4^.

Between 1920 and 1960, genotypes II, III and IV were responsible for most ND outbreaks infecting chickens, waterfowl, and other domestic and wild birds^5^. From 1960 to 1970, outbreaks predominantly included genotypes V and VI and affected ornamental and caged birds^6,7^. Later outbreaks included genotype VI isolates and primarily infected racing pigeons and spread worldwide due to poor biosecurity and biocontainment^7^. The current and ongoing ND outbreak (since 1980s) has been largely caused by genotypes V, VI, VII and XIII infecting mostly chickens^7,8^. New variants of OAVJ continue to emerge despite widespread vaccination against OAVJ in commercial poultry^8^. For surveillance, virological and serological assays are informative about live virus or exposure to OAVJ antigens respectively, but these assays cannot provide information about viral subtypes or pathotypes of OAVJ.

Although multiple viral and host factors can contribute to OAVJ virulence, cleavage of the F protein is essential for virus infectivity. F is synthesized as a F0 precursor and must be cleaved into F1 and F2 for activity^3^. The cleavage happens at a signature cleavage site; in lentogens, it is monobasic (^112^G-R/K-Q-G-R-L^117^) which restricts tropism to tissues that express trypsin- like proteases. In mesogens and velogens, the polybasic cleavage site (^112^R/G/K-R-Q/K-K/R-R-F^117^) is more promiscuous and can be cleaved by a variety of proteases allowing virulent strains to infect systemically^3^. These differences in the fusion cleavage site are the molecular basis for differentiating pathotypes^9^. Currently within the US, all samples suspected to contain OAVJ must be maintained in a cold chain upon collection and shipped overnight to a National Animal Health Laboratory Network (NAHLN) certified state laboratory. Once received at the NAHLN certified state laboratory, samples are processed by trained personnel to isolate RNA which is reverse transcribed and amplified in two individual reactions; the first uses an M gene-based assay to detect OAVJ and the second a F gene-based assay to differentiate between low virulence and virulent OAVJ strains if present.

There is a need for rapid assay(s) that can both detect circulating OAVJ in poultry flocks and differentiate between a virulent strain and a low virulence OAVJ strain, such as the live vaccine strains used, without the need for RNA extraction and purification. Other groups have tested isothermal nucleic acid amplification methods such as loop mediated isothermal amplification (LAMP) and its variants for OAVJ detection^10–15^. LAMP enables rapid, sensitive and specific detection of target templates using isothermal temperature amplification without the need for expensive instrumentation and laboratory setups^16–21^. While LAMP based assays have been extensively demonstrated for multiple human and veterinary pathogens, a clear advantage of these assays was demonstrated during the recent SARS-CoV-2 pandemic. As of 2021, more than 20 LAMP based approaches were developed for detection of SARS-CoV-2, and six of these assays gained FDA emergency use authorization (EUA)^22^. These assays enabled low-cost rapid detection of infected individuals and helped containment in resource limited settings. For OAVJ, three studies have targeted upstream of the fusion cleavage site^11,12,14^, and two studies have targeted downstream of the fusion cleavage site^10,13^, therefore, these assays are unable to differentiate lentogenic and velogenic isolates of OAVJ. Only one study using LAMP has targeted the fusion cleavage site, however, this assay requires two reactions: one RT-LAMP assay targeting a highly conserved region of the HN gene, followed by a second RT-LAMP assay targeting the F gene with a primer binding site across the fusion cleavage site specific for mesogenic and velogenic sequences^15^.

We aimed to improve upon the previously designed OAVJ F gene RT-LAMP assays by targeting the fusion cleavage site using a molecular beacon RT-LAMP (MB-RT-LAMP). The MB-RT-LAMP assay was designed to combine a colorimetric endpoint (for presence of OAVJ) with the ability to discriminate between lentogenic versus velogenic OAVJ pathotypes using a molecular beacon.

## Results

### MB-RT-LAMP amplifies diverse OAVJ genotypes and differentiates pathotypes

**Figure 1:**
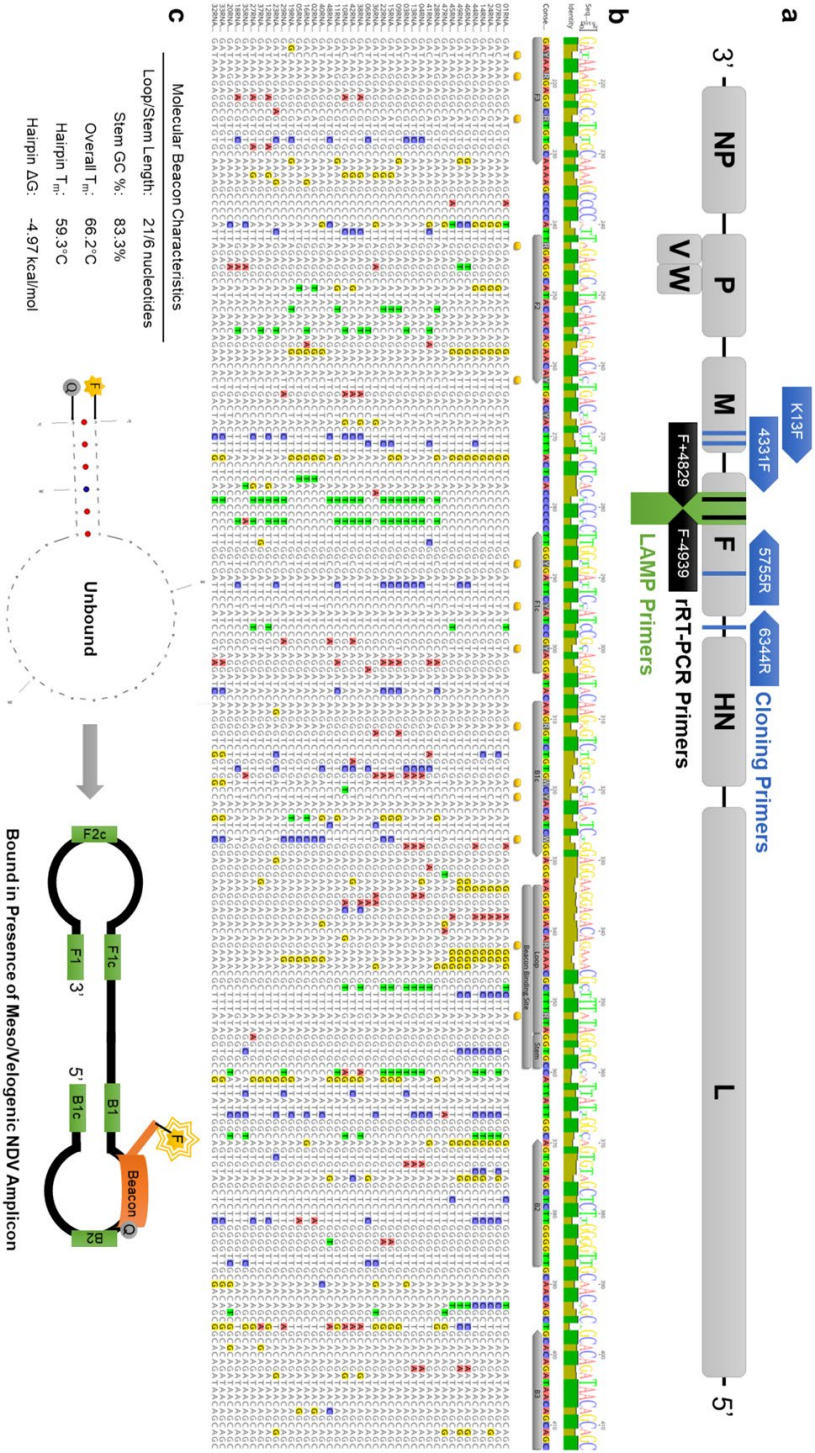
Primer and beacon design schematic. (**a**) Schematic shows the organization of the OAVJ genome with arrows showing location of qRT-PCR, cloning, and LAMP primers. Blue arrows indicate oligonucleotides used for cloning, black arrows indicate oligos used for the current fusion qRT-PCR assay for pathotype discrimination and the green area represents the target used for LAMP primer design. Symbols indicate gene names, and numbers within boxes indicate genomic coordinates. (**b**) MAFFT alignment of 41 diverse partial fusion gene sequences showing fusion cleavage site and MB-RT-LAMP primer and beacon binding sites. Consensus sequence with degree of conservation at each nucleotide position is indicated at the top of the figure. Degenerate bases in primer sequences are indicated by a yellow box below primer binding annotations. Nucleotides varying from the consensus are highlighted in color. (**c**) Characteristics of molecular beacon used are shown. Left panel indicates compositional and thermodynamic characteristics while middle and right panels indicate the mode of action of the molecular beacon used in this manuscript. F = Fluorophore, Q = Quencher, F3 = Outer forward primer, F2 and F1c = two binding sites of Forward Inner Primer (FIP), B1c and B2 = two binding sites of Backward Inner Primer (BIP), B3 = Outer backward primer, F-Loop = Forward loop primer, B-Loop = Backward loop primer, Loop and Stem refer to the loop of the molecular beacon and shared stem binding site.

To design an assay that could detect and differentiate between OAVJ pathotypes, we aligned F gene sequences from 41 diverse OAVJ isolates available from the repository at the USDA Southeast Poultry Research Laboratory (SEPRL) using MAFFT (v. 7.490) to generate a consensus sequence across all fusion sequences. This was used to optimize LAMP primer and molecular beacon design around the fusion cleavage site (Fig. 1a–c and Supplementary Table 1) which is also the site targeted by the OAVJ fusion qRT-PCR assay^9^. Fusion genes from these 41 isolates were reverse transcribed, amplified, and cloned into plasmids for use directly or for in vitro transcription of diverse sequences of pure F gene RNA and details are shown in Supplementary Table 2.

**Figure 2:**
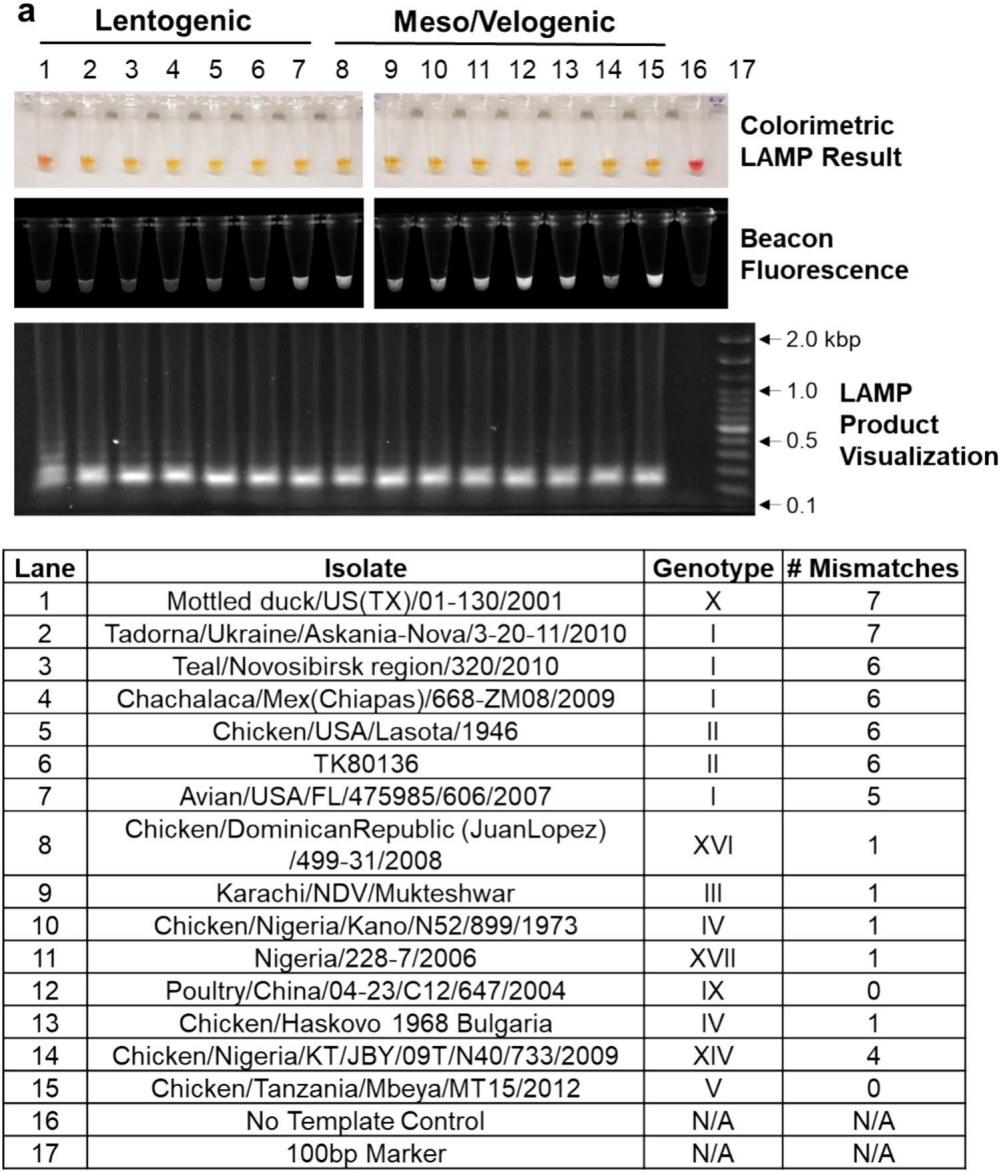

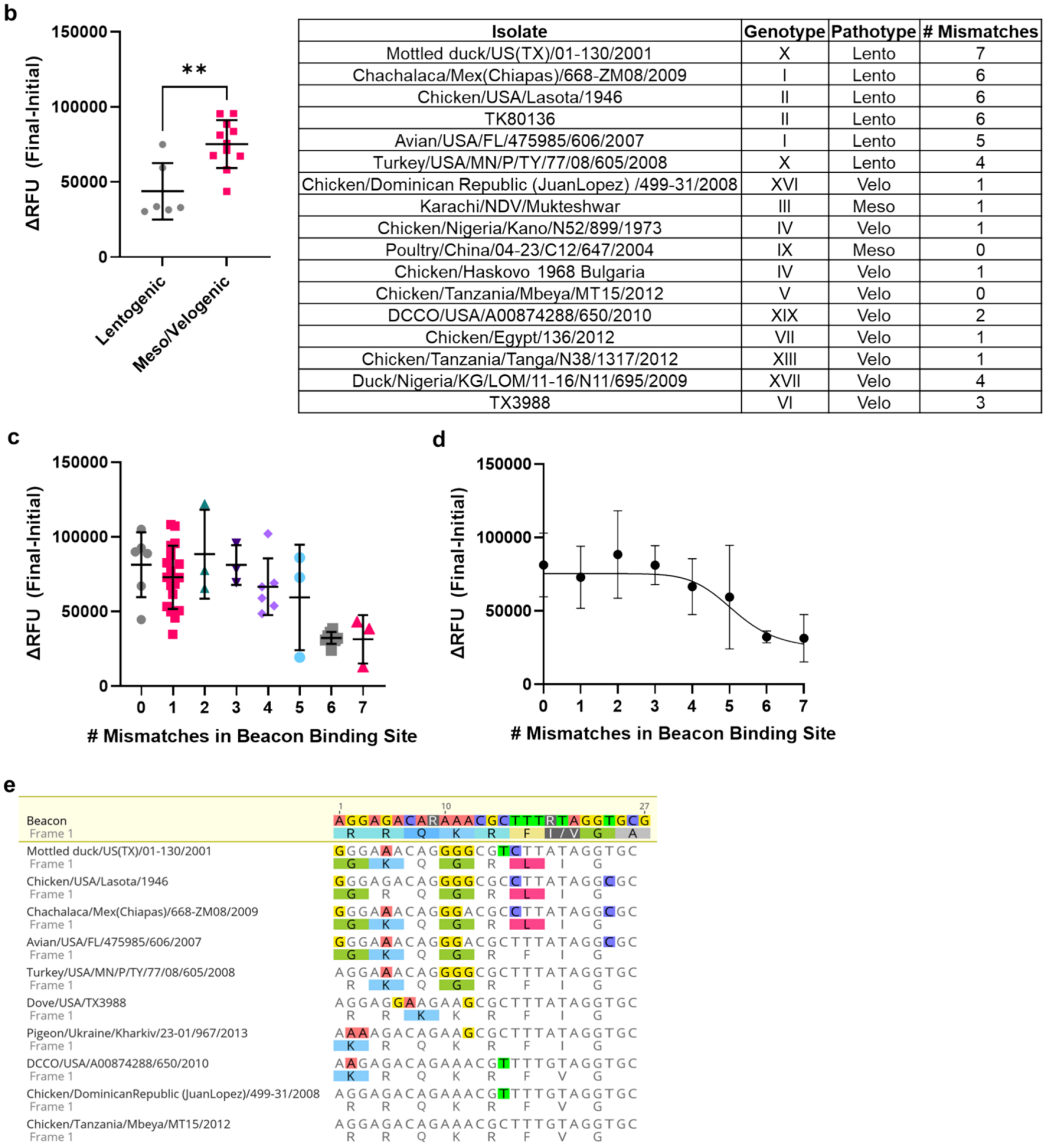
The MB-RT-LAMP assay amplifies and discriminates between different OAVJ fusion plasmids. (**a**) Colorimetric, fluorescent, and gel electrophoresis results (top to bottom) of templates representing diverse genotypes of both lentogenic and meso/velogenic pathotypes are shown. Top panel: MB-RT-LAMP exhibits color change due to amplification with fusion gene plasmid templates for both lentogenic (tubes 1–7) and mesogenic/velogenic fusion genes (lanes 8–15) but not in no template controls (lane 16). Data shown are representative. Middle panel: Panel shows differences in endpoint fluorescence of molecular beacon binding to lentogenic (tubes 1–6) (no binding) versus binding to meso/velogenic (lane 8–15) fusion plasmid templates compared to no beacon signal in no template control. Lane 7 shows misclassification of a lentogenic sequence as velogenic. Bottom panel: Electrophoresed gel showing LAMP products resolved on a 0.8% agarose gel run at 3.6 V/cm in 1X TBE buffer for 1 h and stained with SYBR safe DNA Gel Stain is shown. Table below bottom panel describes samples and controls used. (**b**) The difference in molecular beacon fluorescence binding (ΔRFU_Final–Initial_) for 17 different lentogenic and mesogenic/velogenic fusion templates (listed on right) is shown. Each data point represents the mean ΔRFU from three replicates of each isolate. Error bars represent the mean and standard deviation of each group. Statistical difference of the mean ΔRFU between groups was calculated using a two-tailed unpaired student’s t-test with Welch’s correction (**p = 0.0071). **c)** Graph shows the ΔRFU of each replicate for 17 samples classified by the number of mismatches between each fusion cleavage site and molecular beacon. Error bars indicate mean ± standard deviation for each group. Beacon fluorescence correlated with the number of mismatches with a Pearson correlation coefficient of -0.8699. (**d**) Curve fit of a nonlinear regression to the mean ΔRFU per number of mismatches between the template fusion cleavage site and molecular beacon demonstrates that the best-fit IC_50_ value is 5.139 nucleotide mismatches. (**e**) Select fusion cleavage site nucleotide sequences and amino acid translations representing 0–7 nucleotide mismatches to the molecular beacon (gold highlight). Nucleotide matches are shown in white, and mismatches are highlighted in color.

F gene plasmids generated from diverse OAVJ isolates representing lentogenic, mesogenic, and velogenic pathotypes from 10 different genotypes (I, II, III, IV, V, IX, X, XIV, XVI, and XVII) were chosen for preliminary evaluation of the MB-RT-LAMP assay. MB-RT-LAMP assay end points (colorimetric, fluorescent and electrophoresis of amplification products) were evaluated at 1 h post 57 °C incubation. Reactions were expected to turn yellow in color if positive versus pink for no amplification; data showed that all 15 fusion plasmid templates (representing 10 diverse genotypes) amplified successfully (Fig. 2a top panel, lanes 1–15). No color change was observed for no template control (lane 16). Molecular beacon derived fluorescence was observed only with mesogenic or velogenic templates (Fig. 2a, middle panel, lanes 7–15) except for sample 7 (Avian/USA/FL/475,985/606/2007). This fusion template contains a phenylalanine residue (Phe) at position 117 in the fusion gene possibly misclassifying this sequence as a velogen. Electrophoresis of all reactions (Fig. 2a, bottom panel) showed a ladder pattern typical of LAMP reactions^15^.

Total raw fluorescence data at 1 h endpoint (Fig. 2a, middle panel) showed that the beacon bound to mesogenic/velogenic fusion plasmids but not to lentogenic fusion templates. All mesogenic/velogenic sequences used here (Fig. 2a) contained 0–1 nucleotide mismatches within the beacon binding site (translating to either RRQKR↓F, RRQRR↓F and RRRKR↓F cleavage sites). To quantify this difference in fluorescence and simultaneously expand to more variants of the fusion cleavage site, 17 total fusion templates (representing 13 different genotypes with 0–7 mismatches) (Fig. 2b, table on right) were tested in biological triplicate reactions and the change in relative fluorescence (ΔRFU_Final-Initial_) over the course of the reaction was measured. Lentogenic and mesogenic/velogenic pathotypes showed statistically significant differences in ΔRFU despite the inclusion of two misclassified lentogenic samples (Fig. 2b, *p* = 0.0071). Analysis to establish correlation between mismatches with the typical fusion cleavage site and ΔRFU showed no significant differences up 4 nucleotides (Fig. 2c,d). These did not significantly alter beacon binding signal as shown and include fusion cleavage residue sequences of KRQKR↓F, RRQKR↓F, RRQRR↓F, and RRKKR↓F. Lentogenic fusion cleavage residue sequences that were accurately differentiated included GKQGR↓L and GRQGR↓L. Of note, the two samples that are known lentogenic strains with unique cleavage sites consisting of cleavage residues of RKQGR↓F (Turkey/USA/MN/P/TY/77/08/605/2008: 4 nucleotide mismatches to beacon) or GKQGR↓F (Avian/USA/FL/475,985/606/2007: 5 nucleotide mismatches to beacon) were used in this experiment. Both unique samples were misclassified as velogenic isolates by the MB-RT-LAMP assay, likely due to their cleavage sequences including a phenylalanine at residue 117, which is more commonly associated with velogenic isolates (Fig. 2e).

To balance efficient LAMP amplification with accurate beacon-based discrimination of lentogenic from mesogenic/velogenic sequences, optimization of parameters such as primer concentration, detergents, and endpoint color development was also explored. The LAMP primer and beacon design included degenerate bases to accommodate the sequence diversity in OAVJ fusion genes. Optimal amplification was observed at 1X primer concentration; decreasing (0.5X) primer concentration did not change the detection ability of the assay but increasing the primer concentration to 2X did improve the color change of the assay and reduced time to end point (Supplementary Fig. 1a). Addition of 40–60 mM Guanidine Hydrochloride (GuHCl) to the reaction has been previously shown to increase reaction speed and limit of detection^23^. Similar ten-fold increase in sensitivity (from 2 × 10^3^ copies of LaSota F gene to 2 × 10^2^ copies) was observed upon addition of GuHCl (Supplementary Fig. 1b) but was accompanied by significant reduction in the beacon fluorescence for mesogenic/velogenic reactions (*p* = 0.0011), and therefore the discrimination between pathotypes (Supplementary Fig. 1c,d). Gu-HCl inclusion in the assay was therefore not pursued further.

### MB-RT-LAMP can sensitively detect OAVJ RNA

The MB-RT-LAMP was next repeated with 14 in vitro transcribed (IVT) RNAs (shaded gray in Supplementary Table 2) that represent diverse OAVJ genotypes with a range of mismatches within the molecular beacon binding site. A pilot test showed that the MB-RT-LAMP was able to amplify and discriminate all samples tested (data not shown). IVT RNA from three very diverse samples (DCCO/USA/A00874288/650/2010, Chicken/Tanzania/Tanga/N38/1317/2012, and Chicken/USA/Lasota/1946) having 1, 2 or 6 mismatches respectively, to the beacon were tested using the MB-RT-LAMP at multiple dilutions (10^7^–10^3^ copies) in biological triplicates with a 90-min incubation to allow efficient RT and amplification. Identical samples were also tested using the F gene qRT-PCR^9^ as positive controls.

**Figure 3:**
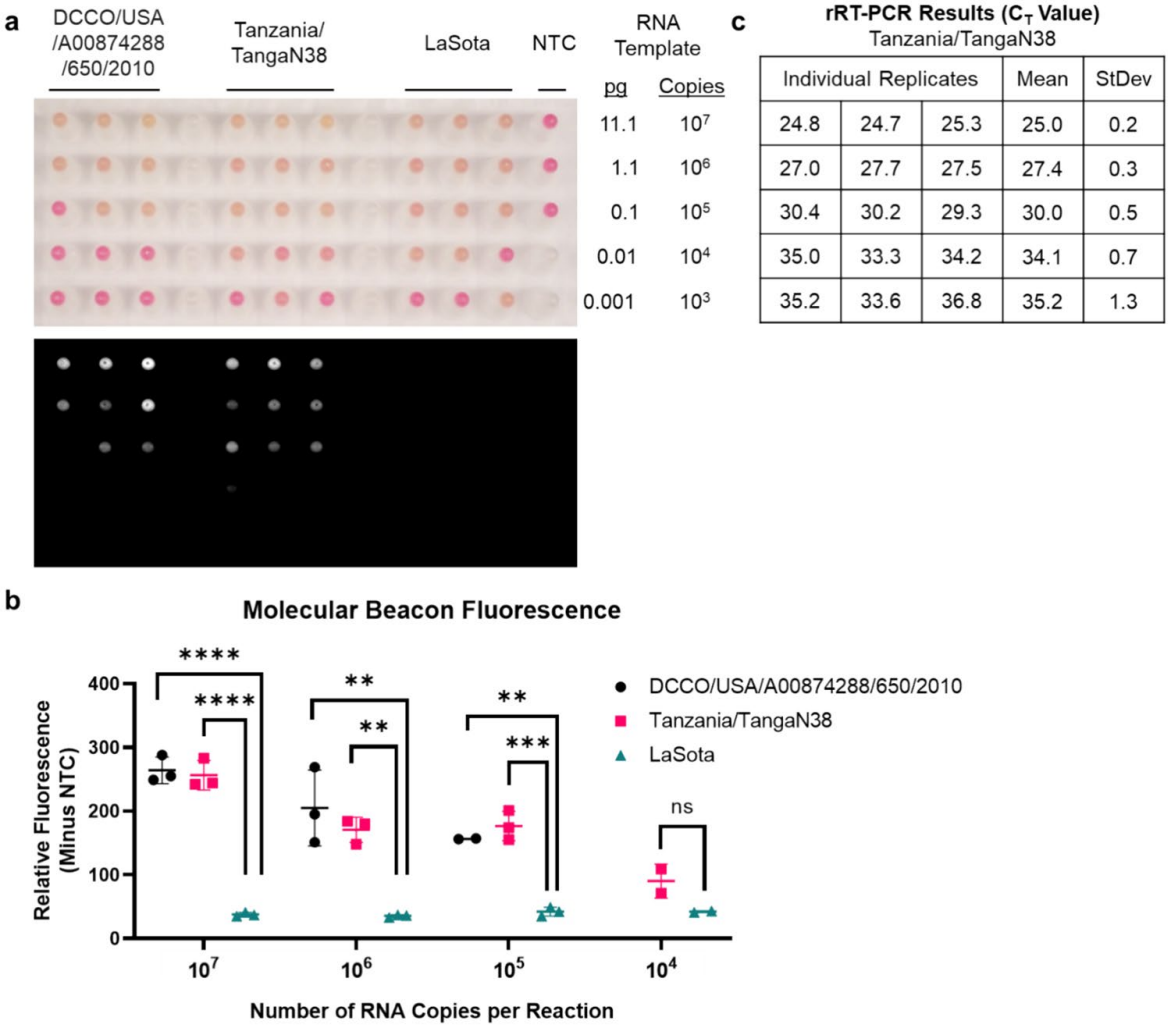
MB-RT-LAMP can sensitively detect OAVJ RNA. (**a**) The MB-RT-LAMP assay can detect a minimum of 10^4^ copies or 0.01 pg of RNA and differentiate samples with as little as 10^5^ copies or 0.1 pg of RNA. The top panel shows the colorimetric endpoint, and the bottom panel shows the fluorescent endpoint of the of the MB-RT-LAMP assay using tenfold serial dilutions of IVT RNA from three samples in triplicate reactions. (**b**) Quantified fluorescence of the molecular beacon demonstrates significant differences (**p < 0.05, ***p < 0.01 and ****p < 0.0001) in brightness between lentogenic and velogenic samples with as little as 10^5^ copies or 0.1 pg of RNA. (**c**) Cycle threshold (Ct) values from the OAVJ F gene qRT-PCR of the Tanzania/TangaN38 IVT RNA serial dilutions run in parallel with the MB-RT-LAMP assay demonstrate that the MB-RT-LAMP assay is nearly as sensitive as the qRT-PCR.

A color change from pink to yellow was observed visually for all dilutions down to 10^5^ copies (0.1 pg) reproducibly for both LaSota and the velogenic viruses (Fig. 3a). A higher dilution of LaSota IVT RNA containing 10^4^ copies (0.01 pg) showed amplification but for only two of the three biological replicates tested. Molecular beacon fluorescence was visually detectable in the two velogenic samples, with no signal in LaSota or NTC containing wells, as hypothesized (Fig. 3b). The differences in fluorescence intensity between the two velogenic strains and lentogenic LaSota strain were statistically significant down to 10^5^ copies of the fusion transcript, suggesting that 10^5^ copies (0.1 pg) was the likely limit of detection (LOD) of the MB-RT-LAMP assay. This was also corroborated by high standard deviation (SD > 0.4) in Ct values at higher dilutions especially around 10^4^ copies in the F gene qRT-PCR assay (Fig. 3c); this suggest that the MB-RT-LAMP is at least a log-fold less sensitive than the 10^4^ copy LOD for the fusion qRT-PCR by Wise et al.^9^.

An increase in ΔRFU was observed when samples were allowed to cool to room temperature prior to data collection and improved discrimination of lentogenic and mesogenic/velogenic sequences (Supplementary Fig. 2). Further, beacon fluorescence trends were found to be instrument agnostic; measurements done with two different equipment, and at different temperatures, showed similar profiles (Supplementary Fig. 2). Statistical comparison showed that beacon fluorescence from the two velogenic samples was significantly greater than from the LaSota IVT RNA samples down to 10^5^ copies per microliter (0.1 pg/μL). This suggests that 10^5^ copies is the reliable LOD for the LAMP assay. These data suggest that the MB-RT-LAMP is about a log-fold less sensitive than the current F gene qRT-PCR at detecting and differentiating OAVJ RNA.

### MB-RT-LAMP is specific for OAVJ RNA

**Figure 4:**
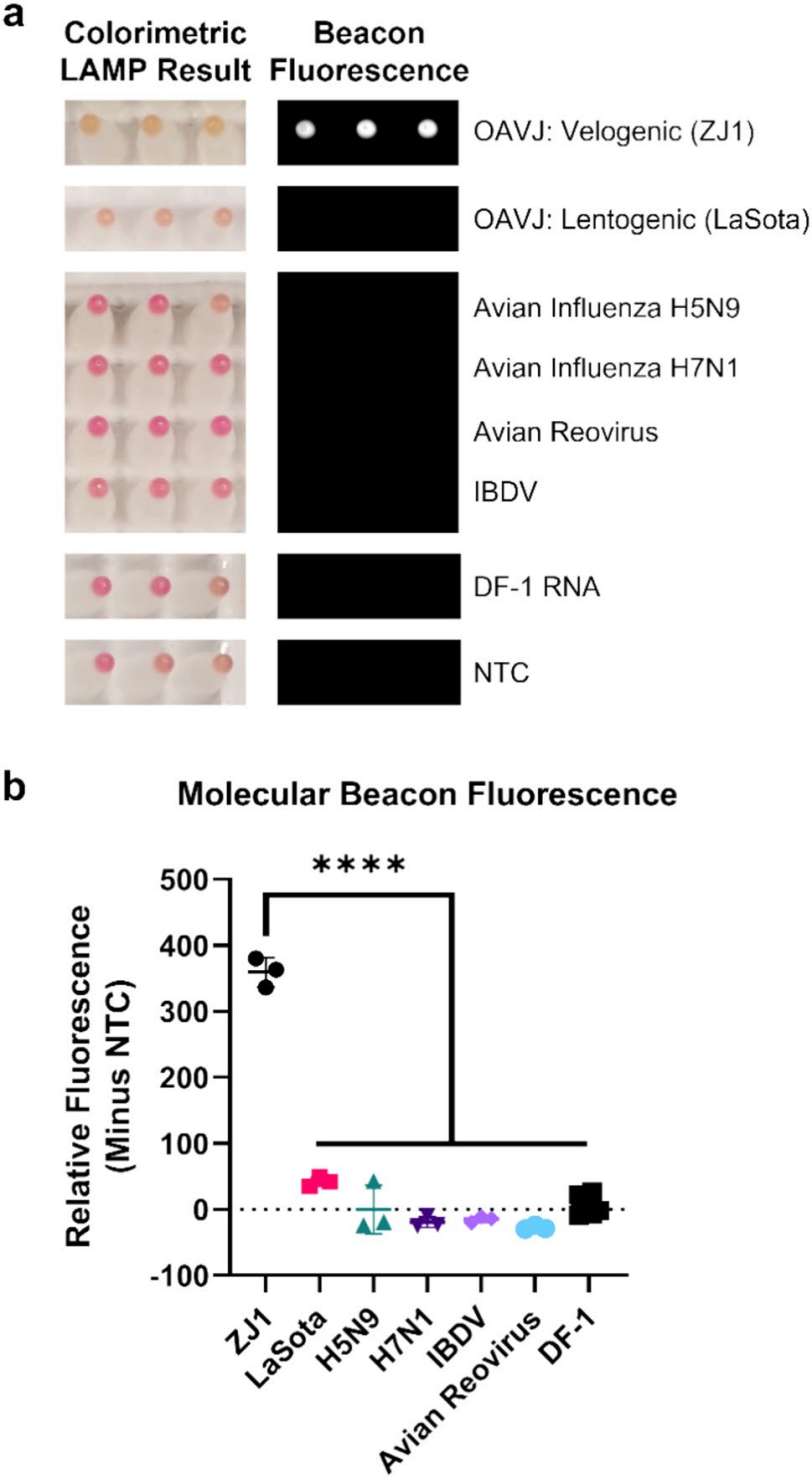
MB-RT-LAMP is specific for OAVJ RNA. (**a**) Left panel shows colorimetric endpoint of MB-RT-LAMP with different RNA templates. A positive color change to yellow is only seen when lentogenic or velogenic OAVJ RNA is present in the reaction, not when RNA from other common poultry pathogens is used. Right panel shows fluorescent endpoint of MB-RT-LAMP reaction, and molecular beacon fluorescence only present from velogenic OAVJ RNA as a template. (**b**) Quantification of molecular beacon fluorescence from samples shown in panel a. Each data point represents the relative fluorescence units (minus fluorescence from NTC reactions) from three replicates of each isolate. Error bars represent the mean and standard deviation of each sample set. Statistical difference of the mean relative fluorescence units between groups was calculated using an ordinary one-way ANOVA with Tukey’s post-hoc correction for multiple comparisons (****p =  < 0.0001).

Testing the OAVJ MB-RT-LAMP assay using purified RNA from other common poultry viruses: avian influenza (H5N9 and H7N1), avian reovirus, infectious bursal disease virus (IBDV), total RNA from chicken fibroblast (DF-1) cells and no template controls did not yield a positive colorimetric signal (Fig. 4a, left panel). A strong beacon signal was observed for control velogenic RNA but not lentogenic OAVJ RNA (Fig. 4a, right panel) confirming the specificity of the LAMP primers and the beacon for OAVJ RNA. Net beacon fluorescence for the velogenic OAVJ was statistically significant (*p* =  < 0.0001) relative to all other RNAs tested (Fig. 4b).

### MB-RT-LAMP can accurately differentiate lentogenic and velogenic OAVJ isolates

**Figure 5:**
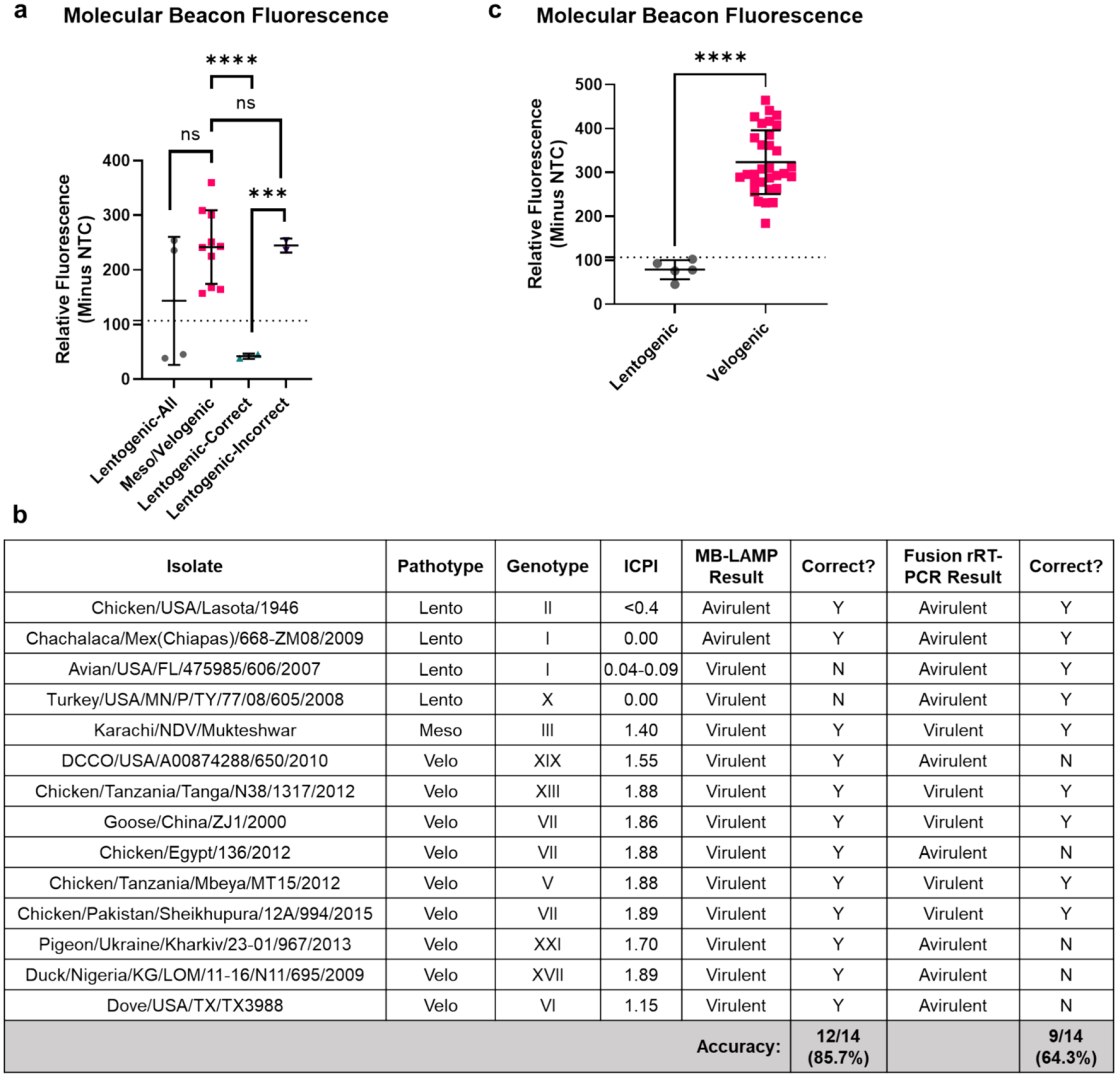
MB-RT-LAMP can accurately differentiate lentogenic and velogenic OAVJ isolates. (**a**) Relative molecular beacon fluorescence from MB-RT-LAMP reactions of all tested IVT RNAs (training set for lentogenic, mesogenic/velogenic sequences and correctly classified or misclassified templates) is plotted. Graph shows the mean molecular beacon fluorescence from biological triplicates for each sample. Molecular beacon signal further separates correctly classified and misclassified lentogenic OAVJ pathotypes (****p =  < 0.0001). Dotted line represents two standard deviations below the mean relative fluorescence units of the mesogenic/velogenic samples. (**b**) Details of the 14 F gene IVT RNA samples used for testing the MB-RT-LAMP accuracy in comparison to the OAVJ F gene qRT-PCR are tabulated. Lento = Lentogenic, Meso = Mesogenic and Velo = Velogenic. ICPI = Intracerebral pathogenicity index, Y = Yes, N = No. Accuracy of the assay is indicated in absolute numbers and percentages. (**c**) Accuracy of lentogenic versus mesogenic/velogenic sequence discrimination by the molecular beacon is graphed. Data shows the mean molecular beacon fluorescence from technical duplicates of correctly classified samples. Statistical significance was computed using an unpaired two-tailed t-test with Welch’s correction (****p =  < 0.0001).

To ensure that the molecular beacon would accurately discriminate velogenic versus lentogenic OAVJ isolates, the MB-RT-LAMP assay was run on a training set of 14 different IVT RNAs to determine the baseline fluorescence threshold (Fig. 5a). Net beacon fluorescence for mesogenic/velogenic RNAs in the training set was higher compared to lentogenic OAVJs but did not appear to be statistically significant. Further analysis revealed that this discrepancy was due to inclusion of two IVT RNA samples misclassified as velogens (Avian/USA/FL/475,985/606/2007 and Turkey/USA/MN/P/TY/77/08/605/2008). Statistical analysis of net beacon fluorescence values following removal of these two samples showed that differences in the mean net relative fluorescence between true lentogens versus true velogens was statistically significant (p =  < 0.0001). The average net beacon fluorescence cutoff for mesogenic/velogenic sequences was calculated as two standard deviations below the mean of mesogenic/velogenic fluorescence, which corresponded to 107.4 relative fluorescence units (Fig. 5a, dashed line).

Since the F qRT-PCR assay is the standard assay used for OAVJ differentiation by NAHLN, we tested if the MB-RT-LAMP could meet the specificity and sensitivity of the current NAHLN F gene qRT-PCR (Fig. 5b). The fusion qRT-PCR assay can correctly discriminate most velogens but is not designed to correctly discriminate the cormorant and pigeon samples^9,24–26^. Results of this testing set for accuracy of the MB-RT-LAMP assay suggested that although some lentogenic sequences were misidentified, the MB-RT-LAMP assay was equally accurate as the OAVJ F gene qRT-PCR at detecting and differentiating mesogenic/velogenic OAVJ sequences (85.7%, Fig. 5b).

Having confirmed that the MB-RT-LAMP assay is sensitive and specific for OAVJ RNA, the analytical specificity was tested with total RNA extracted from 56 virus stocks (15 lentogens and 41 velogens) representing isolates from 15 diverse genotypes (Supplementary Table 3). These numbers were determined using GPower 3.1^27^ to calculate sample size a priori for a two-tailed t-test to determine differences between two independent means with effect size set to 1.0, α error probability to 0.05, and allocation set to 2.0 giving us 81% power achieved. Each sample was analyzed in parallel using three different assays: (1) OAVJ M gene qRT-PCR as a higher sensitivity reference assay to confirm presence of viral RNA in the sample tested^9^, (2) MB-RT-LAMP, and (3) OAVJ F gene qRT-PCR.

The M gene qRT-PCR amplified 15 of the 15 lentogens and 33 of the 41 velogenic samples (Supplementary Fig. 3a and 3b). The MB-RT-LAMP successfully amplified 11 of the 15 (73.3%) lentogens (Supplementary Fig. 3a) and 34 of the 41 (82.9%) velogens (Supplementary Fig. 3b). The molecular beacon gave a false positive signal for 6 of the 11 (54.5%) lentogens (Supplementary Fig. 3a), but correctly identified 31 of the 34 (91.2%) velogens. Fusion qRT-PCR was negative for all 15 (100%) lentogens and correctly identified 30/33 (90.9%) velogens. These data suggest that the MB-RT-LAMP assay approaches the sensitivity of the fusion qRT-PCR assay and equals specificity regarding velogenic sequences.

### Rapid lysis enables direct MB-RT-LAMP testing without RNA purification

**Figure 6:**
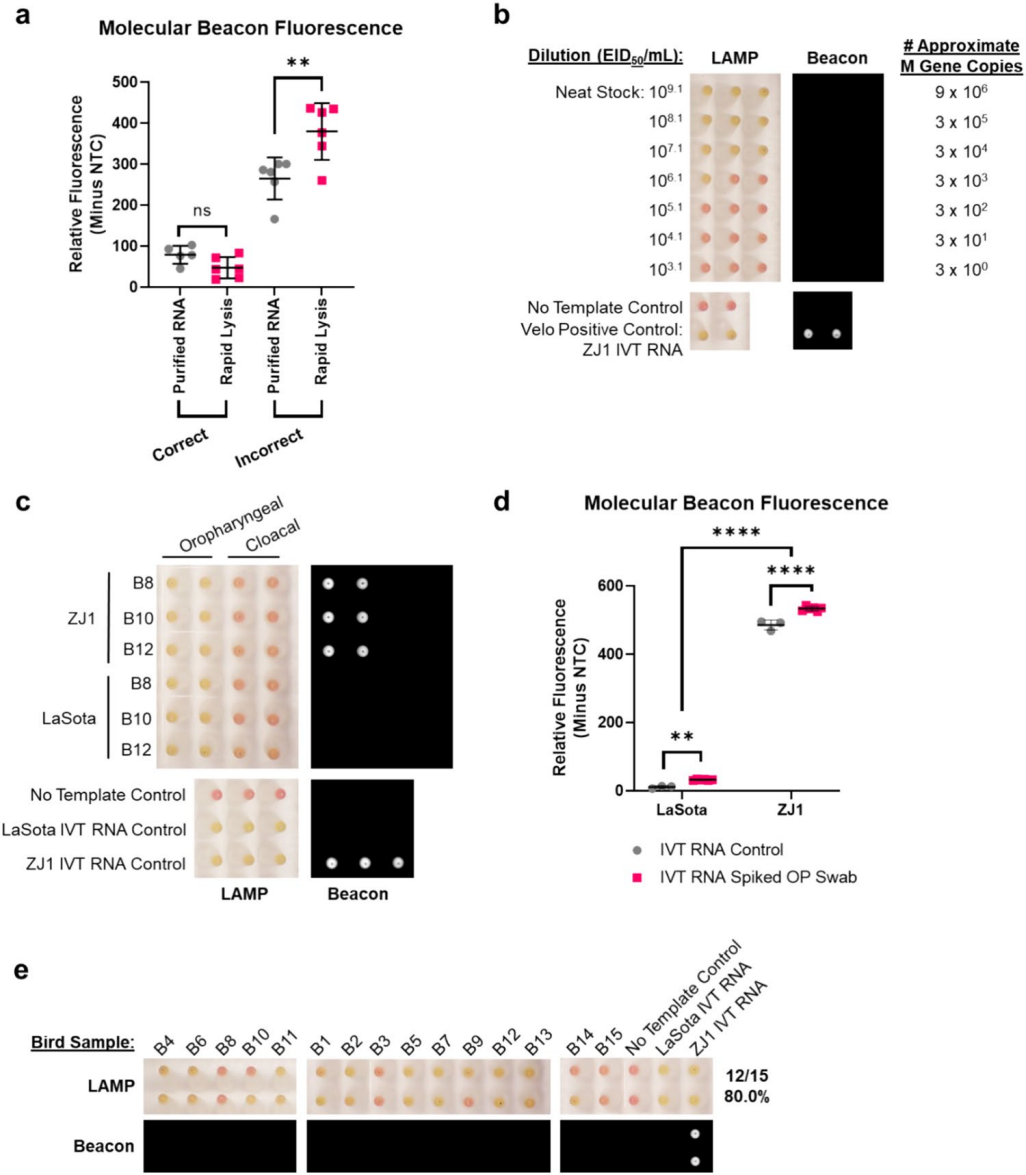
Rapid lysis enables rapid detection of OAVJ RNA from oropharyngeal swabs with MB-RT-LAMP without RNA purification. (**a**) Comparison of relative beacon fluorescence following MB-RT-LAMP using purified RNA or rapid lysed sample for 15 lentogenic isolates of OAVJ. The molecular beacon fluorescence of correctly and incorrectly classified isolates is plotted (ns = not significant.; **p = 0.0094). (**b**) Ten-fold dilutions of LaSota virus (10^9.1^ EID_50_/mL) in BHI, in biological triplicates were tested using rapid lysis MB-RT-LAMP. Left panel shows colorimetric data for triplicates of each dilution and right panel shows corresponding beacon signal if any. Approximate M gene copy numbers are listed in rows. Pink color corresponds to no amplification and orange/yellow corresponds to amplification. Controls are shown at the bottom. (**c**) Left panel shows colorimetric endpoint of velogenic (ZJ1) or lentogenic (LaSota) IVT RNA spiked with oropharyngeal or cloacal swab material following rapid lysis and MB-RT-LAMP from three birds in technical duplicates. Yellow color indicates amplification, pink color indicates no amplification. Right panel shows molecular beacon fluorescence. Controls are shown at bottom. (**d**) Graph shows quantified molecular beacon fluorescence of LaSota and ZJ1 IVT RNA only or IVT RNA in the presence of OP swab material. (**p = 0.0041) and ZJ1 (****p =  < 0.0001), but this did not impact the significant difference in fluorescence between LaSota and ZJ1 (****p =  < 0.0001) as tested by ordinary one-way ANOVA with Tukey’s post-hoc correction for multiple comparisons. (**e**) The rapid lysis to MB-RT-LAMP workflow detects and differentiates OAVJ in OP swabs from 15 birds from a prior study. Top panel indicates colorimetric change; yellow indicates amplification, pink indicates no amplification. Summary of results in numbers and percentages are shown. Bottom panel shows beacon signal.

To overcome the need for RNA isolation, we investigated combinations of different detergents that would yield sufficient viral RNA from swabs without affecting assay sensitivity and/or specificity excluding those that affected the colorimetric readout. For example, Tween-80 (0.1% final concentration) slightly changed the master mix color from dark pink to orange-pink but did not hinder visual discrimination between positive and negative samples (Fig. 6 and Supplementary Fig. 4). Preliminary studies demonstrated that 0.1% sodium dodecyl sulfate (SDS) lysed samples effectively but also reduced beacon discrimination between lentogenic and velogenic RNA sequences. Through empirical testing, we identified that a rapid lysis in 0.1% SDS followed by MB-RT-LAMP including 0.1% Tween-80 and 0.1% tributyl phosphate enabled efficient sample lysis without affecting LAMP sensitivity and beacon specificity (data not shown).

Rapid lysis of swabs dipped in allantoic fluid (AF) stocks of 15 diverse lentogenic OAVJ isolates was next performed as described in methods. Beacon fluorescence was minimal for true lentogens as expected between both purified RNA and rapid lysis. For incorrectly classified lentogens, beacon fluorescence increased from both purified RNA as well as rapid lysed samples (Fig. 6a). The biological limit of rapid lysis MB-RT-LAMP detection was determined by testing ten-fold dilutions of LaSota virus swabs (undiluted titer 10^9.1^ EID_50_/mL) in brain heart infusion (BHI) broth which is typically used as a transport medium for avian swabs. M gene copy numbers were measured in parallel from total RNA isolated from each dilution itself to correlate EID^50^ with M gene copy numbers (which were determined by absolute quantitative PCR as per MIQE guidelines). Rapid lysis released OAVJ RNA amplified in all 3 biological replicates in the 10^7.1^ EID_50_/mL dilution and 1 of the 3 replicates at the 10^6.1^ EID_50_/mL dilution suggesting that the limit of detection of the MB-RT-LAMP assay using this workflow is between 10^6.1^ and 10^7.1^ EID_50_/mL (Fig. 6b). Absolute quantitative M gene qPCR showed that the 10^9.1^ EID^50^ stock contained 9 × 10^6^ copies of M gene thus the rapid lysis MB-RT-LAMP assay approached a limit of detection of 3 × 10^4^ copies of M gene. Beacon fluorescence was absent in all LaSota dilutions, as expected, and was detected in reactions containing the velogenic positive control ZJ1 IVT RNA.

To recapitulate clinical samples from vaccine studies and/or field samples, the rapid lysis MB-RT-LAMP workflow was performed on IVT RNA spiked oropharyngeal (OP) and cloacal (CL) swab samples in BHI from specific pathogen free (SPF) chicks swabbed at 7 days post-hatch from a previous study^28^. Amplification was observed in all reactions despite the presence of OP swab material, with ZJ1 spiked samples also showing positive beacon signal as expected (Fig. 6c left panel) and this difference in beacon fluorescence was statistically significant (Fig. 6d). Spiked samples with CL swab material did not show amplification following lysis and MB-RT-LAMP (Fig. 6c), and lack of amplification was confirmed using agarose gel electrophoresis of MB-RT-LAMP reaction products (Supplemental Fig. 4b).

Finally, the rapid lysis method was evaluated using 15 historical OP swabs from chicks (at 4 days post-hatch) experimentally infected with LaSota virus *in ovo*^28^. The rapid lysis method and MB-RT-LAMP workflow resulted in detection of LaSota RNA in 12 of the 15 samples (80%), with no beacon signal as expected for LaSota RNA (Fig. 6e). Previous evaluation of these swabs demonstrated that virus concentration was between approximately 10^5.5^ and 10^7^ EID_50_/mL, which is in line with the estimated limit of detection of this workflow between 10^6.1^ and 10^7.1^ EID_50_/mL. Overall, these results demonstrate that the rapid lysis method combined with MB-RT-LAMP is effective at detecting and differentiating OAVJ RNA from clinical OP swabs near the limit of detection of the assay, without the need for RNA extraction and purification.

## Discussion

*Orthoavulavirus javaense* (OAVJ), the causative agent of Newcastle disease (ND), displays significant genetic diversity^29^. Pathology of OAVJ is determined by its ability to replicate systemically, which is driven by the cleavage site sequence within the viral F gene. Current molecular diagnostics for OAVJ rely on two qRT-PCRs, the first screening for OAVJ RNA using the M gene primer and probe and the second using a F gene specific primer and probe mixture to determine pathotype^9^. The genetic diversity of class II OAVJs, the need for expensive and often inaccessible instrumentation, consumables and trained personnel can delay response to a ND outbreak and result in significant loss. Isothermal nucleic acid amplification overcomes many of these challenges, however, current isothermal assays for OAVJ either do not differentiate between pathotypes^10,12–14^ or need additional assays^15^, which increases time and costs. The objective of this study was to improve on current isothermal detection technologies for OAVJ by targeting the fusion cleavage site for RT-LAMP and combining that with a molecular beacon that would discriminate between pathotypes in a single tube reaction. Data in this manuscript demonstrate the feasibility of this approach. The MB-RT-LAMP approach yields an assay that is highly specific for OAVJ and enables rapid differentiation of pathotypes. Additionally, by incorporating a rapid lysis buffer step before the assay, we demonstrate that MB-RT-LAMP can be used directly with oropharyngeal swab samples.

Conserved sequences surrounding the F cleavage sequence were targeted for primer binding, and degenerate bases were incorporated to increase the ability to detect diverse OAVJ isolates; a molecular beacon was designed to specifically bind the fusion cleavage site sequence of mesogenic and velogenic sequences (Fig. 1). We demonstrate that this approach can amplify F gene plasmids from a variety of class II genotypes (Fig. 2) via an easy-to-read colorimetric change. Beacon data show that the beacon fluorescence clearly discriminated lentogenic from mesogenic/velogenic sequences with minimal background, and differences in beacon fluorescence were statistically significant (Fig. 2a,b; *p* = 0.0071). Our data identified a small subset of lentogenic viruses that bound the beacon and were misclassified as mesogen/velogens by the MB-RT-LAMP assay. We observed that beacon design in this study can resist up to 4 mismatches within the fusion cleavage site and still correctly differentiate velogenic from lentogenic OAVJs. This is important since it is known that nucleotide mismatches within the molecular beacon binding site can have a major impact on beacon binding, and beacon design plays a pivotal role in mismatch tolerance^30–33^. Data demonstrate that net molecular beacon fluorescence correlated with the number of mismatches within the beacon binding site, and a curve fit of a nonlinear regression suggested that > 5 nucleotide mismatches resulted in no beacon binding (Fig. 2c,d). In-depth studies of the impact of mismatch number and location were not explored as analyses herein were limited to wild-type isolate sequences and other factors can also impact molecular beacon binding.

Using in vitro transcribed RNA dilutions, we demonstrate that the MB-RT-LAMP could reproducibly detect and differentiate lentogenic from mesogenic/velogenic samples with 10^5^ copies of template input. Data at lower copy numbers were encouraging but not reproduced in all replicates (Fig. 3a,b). This is still significant because despite stark differences in the kinetics of amplification, the MB-RT-LAMP limit of detection was within one log of the current F gene-based qRT-PCR assay^9^, the standard assay used for OAVJ differentiation in the NAHLN (Fig. 3c); further improvements in beacon and primer design, additives and crowding reagents may improve on this sensitivity and some were explored. For example, we show that addition of GuHCl improved sensitivity but also dampened beacon signal (Supplementary Fig. 1). Because of fundamental differences in real-time PCR and isothermal LAMP, a direct comparison is not possible although other studies have shown equivalent sensitivities between the two methods^11,15^. Testing the MB-RT-LAMP with a variety of common poultry RNA virus templates clearly established the high specificity of the MB-RT-LAMP assay for OAVJ and the molecular beacon for velogenic OAVJ viruses. (Fig. 4). We further demonstrated that there is a statistically significant difference in the beacon fluorescence between true lentogens and mesogenic/velogenic OAVJs and these agree with F gene  qRT-PCR based differentiation (Fig. 5).

We further tested if the MB-RT-LAMP would correctly identify and differentiate 56 egg-passaged virus stocks or swabs representing 15 different genotypes, multiple bird species, and both lentogenic and velogenic pathotypes (Supplementary Fig. 3, Supplementary Table 3). The MB-RT-LAMP amplified 73% of all lentogens and 83% of all velogens while the beacon correctly identified 91% of mesogenic/velogenic isolates. The MB-RT-LAMP was unable to correctly discriminate three velogenic viruses (Pigeon/Ukraine/Ukromne/3-26-11/2011, Pigeon/Ukraine/Doneck/3/968/2007, and Vulture/Nigeria/PL038-XVII/N47/895/2002–2003). Stocks of the latter two isolates are known to be contaminated with a genotype II strain of OAVJ (Supplementary Table 3); as genotype II sequences are most often lentogenic sequences, LAMP amplicons would not be expected to bind the molecular beacon.

Analyzing the sequences of the lentogenic OAVJs misclassified as velogens by MB-RT-LAMP, we observed that these either contained a phenylalanine at position 117 (Chicken/Georgia/2989/2021, Turkey/USA/MN/P/TY/77/08/605/2008 and Avian/USA/FL/475,985/606/2007) (Supplementary Fig. 3a) or belonged to genotype X Mallard or Northern Pintail isolates (Supplementary Fig. 3a), all of which have 7–8 mismatches within the fusion cleavage site to the molecular beacon (Supplementary Table 3). Computational analysis of molecular beacon binding to these different sequences revealed starkly different heterodimer stabilities (Supplementary Fig. 5). While sequences with zero mismatches had high free energy (ΔG = − 52.27 kcal/mole) and thus high binding stability to the beacon, for sequences with > 4 mismatches or perfect complementarity only within the 5’ or 3’ of the beacon, predicted free energy was low (Supplementary Fig. 5) suggesting that the length of complementary bases on the 3’ end of the beacon beyond the conserved shared-stem binding site contributed to the heteroduplex stability, and therefore misclassification of lentogenic sequences as velogenic. Improvements to beacon design thus need a better understanding of the local solution structure and thermodynamics of binding including the comparison of a conventional beacon design to the shared-stem molecular beacon design used here^34^ and additives that may aid in a better discrimination.

Current workflows for processing OP and CL swabs during surveillance are resource intensive. By testing various detergents either alone or in combination (Supplementary Table 4) we identified a cocktail that enabled rapid release of viral RNA and was compatible with the colorimetric and fluorescent endpoints of MB-RT-LAMP. Data showed that the rapid lysis method gave comparable sensitivity to purified RNA for both lentogenic and velogenic OAVJs (Supplementary Fig. 4a and Fig. 6a). Rapid lysis and MB-RT-LAMP were able to successfully detect 10^6.1^ to 10^7.1^ EID_50_/mL LaSota virus corresponding to approximately 3 × 10^3^ to 3 × 10^4^ copies of template (Fig. 6b).

Clinical/field OP and CL swabs are the standard samples collected during outbreak surveillance. Since the COVID-19 pandemic made it challenging to obtain and test clinical/field OP and CL swabs, we demonstrated (Fig. 6) that MB-RT-LAMP successfully amplified and differentiated OP swabs spiked with LaSota or ZJ1 IVT RNA. Inhibitory compounds present in CL swabs have been documented to inhibit nucleic acid amplification^35^ and require additional processing; the MB-RT-LAMP was unsuccessful with CL swabs most likely due to these inhibitors and we are currently investigating modifications to the workflow that would allow us to remove these in the rapid lysis buffer (Fig. 6c and Supplementary Fig. 4b). Finally, MB-RT-LAMP testing of OP swabs from 15 chicks at 4 days post-hatch that had been experimentally infected with LaSota virus *in ovo* demonstrated 80% successful amplification (Fig. 6e). This was significant as the samples were known to be near the limit of detection of the MB-RT-LAMP assay.

Data shown in this manuscript supports the hypothesis that the rapid lysis-MB-RT-LAMP workflow performs well with current sample collection and storage practices used in avian diagnostics, so no adaptation would be needed for implementation of this assay in practice. Since the rapid lysis incubation is only for a few minutes, it may not be suitable for complex matrices such as tissues unless viral loads are very high. It is important to note that the LOD for the MB-RT-LAMP was a log lower than conventional qRT-PCR; thus, the assay may be more suited to preliminary screens from large number of samples so that “positive” ones can be validated by F gene qRT-PCR. A significant advantage of the assay lies in the simplicity of sample processing which can be done in the sample collection tube itself followed by a single tube detection and differentiation workflow making it more suited to test samples in the field with the inclusion of a simple light box for visualization of molecular beacon fluorescence^32,36^.

LAMP-based assays have been developed and used for multiple pathogens, most notably with the recent COVID-19 pandemic. A single tube assay like MB-RT-LAMP can complement screening with qRT-PCR, especially in resource limited settings or can be used as an alternative when infrastructure is not available. Since LAMP master mixes can be lyophilized and stored at room temperature, the need for a cold chain is overcome, greatly reducing costs. For example, assuming operator costs as equal and instrumentation procurement costs as a one-time investment, the current qRT-PCR assay method costs ~ $10-$12 per sample and needs a minimum of 3 h to go from sample to PCR data. In contrast, the MB-RT-LAMP assay costs approximately $5 per sample and can be completed in under two hours with less use of consumables.

Outbreak management requires effort on multiple fronts for gathering precise information. In the case of ND, observation of clinical signs, surveillance for the presence of OAVJ RNA in different areas, epidemiologic analyses, and follow-up assessment of the virus causing the outbreak to determine genotype and complete genome sequence are all crucial steps for containing the spread of OAVJ^37^. The ability to rapidly and accurately detect the presence of OAVJ RNA is critical. The studies herein demonstrate the development of a MB-RT-LAMP assay that can detect and differentiate velogenic OAVJ sequences with similar specificity, and within one-log sensitivity of, the current two-assay qRT-PCR method, using only one assay. Further, the use of a rapid lysis method removes the need for RNA isolation before completing the MB-RT-LAMP assay.

## Methods

### LAMP primer design

The F gene sequences for 41 diverse class II genotype OAVJs were obtained from GenBank (Supplementary Table 2), then aligned using MAFTT v7.490 in Geneious Prime v2023.0.1 (Biomatters, Inc., USA, www.geneious.com). Primers were designed using PrimerExplorer V5 (Eiken Chemical Co., LTD., Japan, http://primerexplorer.jp/lampv5e/index.html) targeting the most conserved regions flanking the fusion cleavage site including degenerate bases as needed, specifically for the fusion cleavage site to be located on the B-loop of the dumbbell structure. Three different LAMP primer sets were evaluated before shortlisting the primers used in this manuscript (Supplementary Table 1). For the Forward Inner Primer (FIP) and Backward Inner Primer (BIP), the two binding sequences (F2/F1c and B1c/B2) were connected using a 4-nucleotide thymine linker, which has been shown to improve LAMP efficiency by aiding in loop formation^38^. Primer sets were evaluated using the OligoAnalyzer™ Tool (Integrated DNA Technologies, Inc., USA)^39^. Primers were reconstituted in molecular grade water to 100 µM stock concentration. Working stocks at 10X concentration in water contained 16 µM of FIP and BIP, 2 µM of F3 and B3, and 4 µM of F-Loop and B-Loop primers.

### Molecular beacon design

Four different beacon approaches were evaluated during beacon design and optimization before finalizing a shared-stem design. Final LAMP primers were designed around the fusion cleavage site within the B-loop of the dumbbell with maximal distance between B1c and B2 to allow for binding of the B-loop primer and/or a molecular beacon during amplification for pathotype differentiation. These parameters were adapted from prior LAMP and molecular literature focusing on assay temperature, overall beacon Tm, beacon hairpin Tm, hairpin ΔG, loop and stem lengths, stem G/C content, sequence mismatch tolerances, and predicted secondary structure of the sequence. The molecular beacon was commercially synthesized (Millipore Sigma, USA) with a 5’ reporter of hexachlorofluorescein (HEX) and 3’ quencher of Black Hole Quencher 1 (BHQ1), reconstituted in molecular grade water at 100 μM, then diluted to a 10 μM working stock.

### Generation of OAVJ F gene plasmids

Stocks of OAVJ viruses used in this study were grown in 9–11 day old SPF embryonated chicken eggs as per standard protocol^4^. Total RNA from virus stocks was isolated using Trizol LS and/or MagMAX™-96 AI/ND Viral RNA Isolation Kit (ThermoFisher Scientific, USA) as per standard protocol. Total RNA was quantified and equal amounts were used for cDNA synthesis using Maxima H- reverse transcriptase (ThermoFisher Scientific, USA) as per manufacturer's recommendations. The OAVJ F gene was amplified using proofreading Q5 2X Master Mix (New England Biolabs, MA, USA) with the primers 4331F and 6344Rn, K13/4237F and 6344Rn (Pigeon/Ukraine/Kharkiv/23-01/967/2013 isolate), or DR4297F and DR5100R (Chicken/Dominican Republic (JuanLopez) /499-31/2008 isolate) (NEB, MA, USA). Amplification conditions included initial denaturation at 98 °C for 30 s, then 25 cycles of 98 °C for 10 s, annealing at 64 °C for 30 s and extension at 72 °C for 1 min, with a final extension step at 72 °C for 2 min in a T100 PCR machine (BioRad, USA). Amplicons were resolved in 0.8% agarose in 1X TBE at 3.6 V/cm for 1 h, gel purified using the Monarch DNA Gel Extraction Kit (New England Biolabs, MA, USA), then ligated into pMiniT 2.0 vector (PCR cloning kit; New England Biolabs, MA, USA) as per manufacturer's protocol. Positive clones were screened by colony PCR using the kit forward primer and an F gene internal primer, 5755R, with Taq 2X Master Mix (New England Biolabs, MA, USA), using the following conditions: initial denaturation at 95 °C for 2 min, then 25 cycles of 95 °C for 30 s, annealing at 56 °C for 30 s and extension at 68 °C for 1 min and 45 s, with a final extension step at 68 °C for 5 min. Plasmid minipreps of positive clones were prepared as per Monarch Plasmid Miniprep Kit (New England Biolabs, MA, USA), quantified and verified by Sanger sequencing following manufacturer recommendations for the BigDye™ Terminator v1.1 Cycle Sequencing Kit (ThermoFisher Scientific, USA) using an Applied Biosystems 3730XL DNA Analyzer (ThermoFisher Scientific, USA). Primers used in this study are shown in Supplementary Table 1.

### Generation of Control RNA

Freshly grown single colonies of individual F gene clones were grown in primary Luria–Bertani (LB) broth containing 50 µg/mL ampicillin for 8 h at 37 °C with shaking at 250 rpm. Primary cultures were used to inoculate secondary cultures for plasmid midi preps and plasmid DNA was isolated as per E.Z.N.A.® Plasmid DNA Midi Kit (Omega Bio-tek, GA, USA) following manufacturers recommendations. Plasmid DNA was eluted in 1 mL of elution buffer and quantified. Plasmid DNAs were linearized at a single cut site using PacI (New England Biolabs, MA, USA), gel purified, quantified and then used as templates for in vitro transcription (IVT) of single stranded RNA using the HiScribe® SP6 RNA Synthesis Kit (New England Biolabs, MA, USA) following manufactures recommendations. Briefly, 300–500 ng of PacI linearized F gene plasmid was used as template in a 25µL reaction consisting of SP6 reaction buffer, dNTPs, and SP6 RNA polymerase mix. This reaction mixture was incubated at 37 °C for 2 h, then diluted with 25 µL nuclease free water. Template DNA was removed by the addition of 4 units of DNaseI and incubation at 37 °C for 30 min. To ensure plasmid template had been removed, 5% of the total reaction volume was used as template in PCR using Luna® Universal qPCR Master Mix (New England Biolabs, MA, USA) with primers flanking the fusion cleavage site (F + 4829 and F-4939). IVT RNA was quantified using the Qubit RNA HS (High Sensitivity) Assay Kit (ThermoFisher Scientific, USA), then stored at − 80 °C in single-use aliquots.

### M and F gene qRT-PCR

The M gene qRT-PCR is used for detection of nearly all OAVJ strains, and is highly sensitive^9^. The F gene qRT-PCR is specific for virulent OAVJ strains^9^. Both qRT-PCR tests were performed using TaqMan probes with the AgPath-ID one-step RT-PCR Kit (Ambion) as previously described^9^ and QuantStudio™ 5 Real-Time PCR Instrument using the fast setting (ThermoFisher Scientific, USA). For the M gene qRT-PCR, the reverse transcription step was carried out at 45 °C for 10 min following by reverse transcriptase inactivation at 95 °C for 10 min. PCR cycling included 40 cycles of 95 °C for 10 s, 56 °C for 30 s, and 72 °C for 10 s. The F gene qRT-PCR was run using the same conditions, except the annealing temperature for each cycle was 58 °C. For direct comparison to MB-RT-LAMP, these assays were run in parallel using the same IVT RNA samples, or total extracted RNA samples as prepared for the MB-RT-LAMP assay.

### MB-RT-LAMP assay

Reactions were carried out in 10 μL volumes and contained WarmStart® Colorimetric LAMP 2X Master Mix with UDG (Catalog # M1804, NEB, USA), 1X primer mix, 0.5 μM molecular beacon, molecular biology grade water, and template. Reactions were mixed, then incubated at 57 °C for 60–90 min in a QuantStudio™ 5 Real-Time PCR Instrument (ThermoFisher Scientific, USA), with fluorescence measured every 30 s. Colorimetric results were visually inspected. Fluorescent endpoints were quantified using the VIC channel on the QuantStudio™ 5 Real-Time PCR Instrument (ThermoFisher Scientific, USA), and the increase in fluorescence over the course of the reaction was calculated by subtracting the initial value of fluorescence from the final measured value after incubation(ΔRFU_Final–Initial_). Fluorescence was also measured using a BioTek Synergy HTX Multimode Microplate Reader (Agilent, CA, USA) using λ540/35_Ex_/λ590/20_Em_ with automatic gain. Background fluorescence was removed by subtracting the average value of the fluorescence from the no template control wells (Relative Fluorescence Minus NTC).

### Rapid lysis method for MB-RT-LAMP

A PurFlock Ultra 6″ Sterile Mini-tip Flock Swab (SKU#: 25-3316-U, Puritan Medical Products, ME, USA) was dipped into clarified allantoic fluid from egg passaged viruses, or virus stock diluted in brain heart infusion (BHI) broth to collect virus material. The swab was then placed into a 250 μl solution of 0.1% sodium dodecyl sulfate (SDS) in molecular grade water, spun 5 times and then incubated for 2 min. One microliter of the lysis solution was used as template for the MB-RT-LAMP assay (10% of total reaction volume). The MB-RT-LAMP assay master mix was supplemented with Tween-80 and tributyl phosphate at a final concentration of 0.1% (v/v) each.

### Statistics

All statistical analyses were completed using GraphPad Prism ver. 9.6.4 (build 681). Statistical analyses were carried out using two-tailed unpaired T-test with Welch’s correction, or an ordinary one-way ANOVA with Tukey’s post-hoc test for multiple comparisons. Data represent the mean ± standard deviation of biological triplicates or technical duplicates.

### Ethics statement

No animal experiments were performed in this study. Only swabs collected in a prior study and stored on site were used for experimental validation. Hence, AUP related details are not applicable.

### Supplementary Information

Supplementary Information.

## Data Availability

All raw data pertaining to this manuscript is being deposited to the National Agricultural data library. Data sets used and analyzed in this study are included in this article, supplementary materials and are also available from corresponding author on reasonable request.

## Abbreviations

NDV: Newcastle disease virus
Velo: ﻿Velogenic
Lento: ﻿Lentogenic
F: Fusion
LAMP: Loop mediated isothermal amplification
MB: Molecular beacon

## References

[1] Butt, S. L. *et al.* Tropism of Newcastle disease virus strains for chicken neurons, astrocytes, oligodendrocytes, and microglia. *BMC Vet. Res.* **15**, 317. 10.1186/s12917-019-2053-z (2019). 31484573 10.1186/s12917-019-2053-z PMC6727330

[2] Diel, D. G. *et al.* Complete genome and clinicopathological characterization of a virulent Newcastle disease virus isolate from South America. *J. Clin. Microbiol.* **50**, 378–387. 10.1128/JCM.06018-11 (2012). 22135263 10.1128/JCM.06018-11 PMC3264182

[3] Dortmans, J. C., Koch, G., Rottier, P. J. & Peeters, B. P. Virulence of Newcastle disease virus: What is known so far?. *Vet. Res.* **42**, 122. 10.1186/1297-9716-42-122 (2011). 22195547 10.1186/1297-9716-42-122 PMC3269386

[4] WOAH. *OIE manual of diagnostic tests and vaccines for terrestrial animals* 964–983 (World Organization for Animal Health (WOAH), 2021).

[5] Wang, J. *et al.* Genomic characterizations of six pigeon paramyxovirus type 1 viruses isolated from live bird markets in China during 2011 to 2013. *PLoS One* **10**, e0124261. 10.1371/journal.pone.0124261 (2015). 25928057 10.1371/journal.pone.0124261 PMC4415766

[6] Pearson, G. L. & McCann, M. K. The role of indigenous wild, semidomestic, and exotic birds in the epizootiology of velogenic viscerotropic Newcastle disease in southern California, 1972–1973. *J. Am. Vet. Med. Assoc.* **167**, 610–614 (1975). 1176357

[7] Walker, J. W., Heron, B. R. & Mixson, M. A. Exotic Newcastle disease eradication program in the United States. *Avian. Dis.* **17**, 486–503 (1973). 4748340 10.2307/1589147

[8] Herczeg, J. *et al.* Two novel genetic groups (VIIb and VIII) responsible for recent Newcastle disease outbreaks in Southern Africa, one (VIIb) of which reached Southern Europe. *Arch. Virol.* **144**, 2087–2099. 10.1007/s007050050624 (1999). 10603164 10.1007/s007050050624

[9] Wise, M. G. *et al.* Development of a real-time reverse-transcription PCR for detection of newcastle disease virus RNA in clinical samples. *J. Clin. Microbiol.* **42**, 329–338. 10.1128/JCM.42.1.329-338.2004 (2004). 14715773 10.1128/JCM.42.1.329-338.2004 PMC321685

[10] Pham, H. M., Nakajima, C., Ohashi, K. & Onuma, M. Loop-mediated isothermal amplification for rapid detection of Newcastle disease virus. *J. Clin. Microbiol.* **43**, 1646–1650. 10.1128/JCM.43.4.1646-1650.2005 (2005). 15814979 10.1128/JCM.43.4.1646-1650.2005 PMC1081312

[11] Li, Q. *et al.* An improved reverse transcription loop-mediated isothermal amplification assay for sensitive and specific detection of Newcastle disease virus. *Arch. Virol.* **154**, 1433–1440. 10.1007/s00705-009-0464-z (2009). 19649763 10.1007/s00705-009-0464-z

[12] Tian, B. *et al.* Rapid newcastle disease virus detection based on loop-mediated isothermal amplification and optomagnetic readout. *ACS Sens.* **1**(10), 1228–1234. 10.1021/acssensors.6b00379 (2016). 10.1021/acssensors.6b00379

[13] Liang, R. *et al.* Development of a TaqMan loop-mediated isothermal amplification assay for the rapid detection of pigeon paramyxovirus type 1. *Arch. Virol.* **166**, 1599–1605. 10.1007/s00705-021-04963-w (2021). 33755802 10.1007/s00705-021-04963-w PMC7986176

[14] Selim, K., Adel, A., Eid, S. & Shahein, M. Development of real time reverse transcription loop-mediated isothermal amplification assay for rapid detection of genotype VII of Newcastle disease viruses. *Br. Poult. Sci.* **63**, 864–870. 10.1080/00071668.2022.2094219 (2022). 35791891 10.1080/00071668.2022.2094219

[15] Song, H. S., Kim, H. S., Kim, J. Y., Kwon, Y. K. & Kim, H. R. The development of novel reverse transcription loop-mediated isothermal amplification assays for the detection and differentiation of virulent newcastle disease virus. *Int. J. Mol. Sci.* **24**, 13847. 10.3390/ijms241813847 (2023). 37762149 10.3390/ijms241813847 PMC10531153

[16] Das, D., Lin, C. W. & Chuang, H. S. LAMP-based point-of-care biosensors for rapid pathogen detection. *Biosensors (Basel)* **12**, 1068. 10.3390/bios12121068 (2022). 36551035 10.3390/bios12121068 PMC9775414

[17] Garg, N., Ahmad, F. J. & Kar, S. Recent advances in loop-mediated isothermal amplification (LAMP) for rapid and efficient detection of pathogens. *Curr. Res. Microb. Sci.* **3**, 100120. 10.1016/j.crmicr.2022.100120 (2022). 35909594 10.1016/j.crmicr.2022.100120 PMC9325740

[18] Mannier, C. & Yoon, J. Y. Progression of LAMP as a result of the COVID-19 pandemic: Is PCR finally rivaled?. *Biosensors (Basel)* **12**, 492. 10.3390/bios12070492 (2022). 35884295 10.3390/bios12070492 PMC9312731

[19] Pu, R. *et al.* The screening value of RT-LAMP and RT-PCR in the diagnosis of COVID-19: Systematic review and meta-analysis. *J. Virol. Methods* **300**, 114392. 10.1016/j.jviromet.2021.114392 (2022). 34856308 10.1016/j.jviromet.2021.114392 PMC8629515

[20] Sharma, S., Singh, J., Sen, A. & Anvikar, A. R. Multiplex loop mediated isothermal amplification (m-LAMP) as a point of care technique for diagnosis of malaria. *J. Vector Borne Dis.* **59**, 29–36. 10.4103/0972-9062.331409 (2022). 35708401 10.4103/0972-9062.331409

[21] Velayudhan, B. T. & Naikare, H. K. Point-of-care testing in companion and food animal disease diagnostics. *Front. Vet. Sci.* **9**, 1056440. 10.3389/fvets.2022.1056440 (2022). 36504865 10.3389/fvets.2022.1056440 PMC9732271

[22] Moehling, T. J., Choi, G., Dugan, L. C., Salit, M. & Meagher, R. J. LAMP diagnostics at the point-of-care: Emerging trends and perspectives for the developer community. *Expert Rev. Mol. Diagn.* **21**, 43–61. 10.1080/14737159.2021.1873769 (2021). 33474990 10.1080/14737159.2021.1873769

[23] Zhang, Y. *et al.* Enhancing colorimetric loop-mediated isothermal amplification speed and sensitivity with guanidine chloride. *Biotechniques* **69**, 178–185. 10.2144/btn-2020-0078 (2020). 32635743 10.2144/btn-2020-0078

[24] Diel, D. G. *et al.* Characterization of Newcastle disease viruses isolated from cormorant and gull species in the United States in 2010. *Avian. Dis.* **56**, 128–133. 10.1637/9886-081111-Reg.1 (2012). 22545538 10.1637/9886-081111-Reg.1

[25] Kim, L. M., Afonso, C. L. & Suarez, D. L. Effect of probe-site mismatches on detection of virulent Newcastle disease viruses using a fusion-gene real-time reverse transcription polymerase chain reaction test. *J. Vet. Diagn. Invest.* **18**, 519–528. 10.1177/104063870601800601 (2006). 17121078 10.1177/104063870601800601

[26] Kim, L. M. *et al.* Biological and phylogenetic characterization of pigeon paramyxovirus serotype 1 circulating in wild North American pigeons and doves. *J. Clin. Microbiol.* **46**, 3303–3310. 10.1128/JCM.00644-08 (2008). 18716227 10.1128/JCM.00644-08 PMC2566108

[27] Faul, F., Erdfelder, E., Lang, A. G. & Buchner, A. G*Power 3: A flexible statistical power analysis program for the social, behavioral, and biomedical sciences. *Behav. Res. Methods* **39**, 175–191. 10.3758/bf03193146 (2007). 17695343 10.3758/bf03193146

[28] Dimitrov, K. M. *et al.* Novel Recombinant Newcastle Disease Virus-Based In Ovo Vaccines Bypass Maternal Immunity to Provide Full Protection from Early Virulent Challenge. *Vaccines (Basel)* **9** (2021). 10.3390/vaccines9101189 10.3390/vaccines9101189 PMC8538074 34696297

[29] Dimitrov, K. M. *et al.* Updated unified phylogenetic classification system and revised nomenclature for Newcastle disease virus. *Infect. Genet. Evol.* **74**, 103917. 10.1016/j.meegid.2019.103917 (2019). 31200111 10.1016/j.meegid.2019.103917 PMC6876278

[30] Bonnet, G., Tyagi, S., Libchaber, A. & Kramer, F. R. Thermodynamic basis of the enhanced specificity of structured DNA probes. *Proc. Natl. Acad. Sci. USA* **96**, 6171–6176. 10.1073/pnas.96.11.6171 (1999). 10339560 10.1073/pnas.96.11.6171 PMC26854

[31] Varona, M. & Anderson, J. L. Visual detection of single-nucleotide polymorphisms using molecular beacon loop-mediated isothermal amplification with centrifuge-free DNA extraction. *Anal. Chem.* **91**, 6991–6995. 10.1021/acs.analchem.9b01762 (2019). 31099243 10.1021/acs.analchem.9b01762

[32] Sherrill-Mix, S., Van Duyne, G. D. & Bushman, F. D. Molecular beacons allow specific RT-LAMP detection of B.1.1.7 variant SARS-CoV-2. *J. Biomol. Tech.* **32**, 98–101. 10.7171/jbt.21-3203-004 (2021). 35027867 10.7171/jbt.21-3203-004 PMC8730518

[33] Varona, M., Eitzmann, D. R., Pagariya, D., Anand, R. K. & Anderson, J. L. Solid-phase microextraction enables isolation of BRAF V600E circulating tumor DNA from human plasma for detection with a molecular beacon loop-mediated isothermal amplification assay. *Anal. Chem.* **92**, 3346–3353. 10.1021/acs.analchem.9b05323 (2020). 31950824 10.1021/acs.analchem.9b05323 PMC7155775

[34] Tsourkas, A., Behlke, M. A. & Bao, G. Structure-function relationships of shared-stem and conventional molecular beacons. *Nucleic Acids Res.* **30**, 4208–4215. 10.1093/nar/gkf536 (2002). 12364599 10.1093/nar/gkf536 PMC140536

[35] Das, A., Spackman, E., Pantin-Jackwood, M. J. & Suarez, D. L. Removal of real-time reverse transcription polymerase chain reaction (RT-PCR) inhibitors associated with cloacal swab samples and tissues for improved diagnosis of Avian influenza virus by RT-PCR. *J. Vet. Diagn. Invest.* **21**, 771–778. 10.1177/104063870902100603 (2009). 19901277 10.1177/104063870902100603

[36] Sherrill-Mix, S. *et al.* Detection of SARS-CoV-2 RNA using RT-LAMP and molecular beacons. *Genome Biol.* **22**, 169. 10.1186/s13059-021-02387-y (2021). 34082799 10.1186/s13059-021-02387-y PMC8173101

[37] USDA-APHIS. Epidemiologic Analyses of Virulent Newcastle Disease in Poultry in California, March 2021. (Fort Collins, CO., 2021).

[38] Hwang, J. *et al.* Detection of coat protein gene of nervous necrosis virus using loop-mediated isothermal amplification. *Asian Pac. J. Trop. Med.* **9**, 235–240. 10.1016/j.apjtm.2016.01.035 (2016). 26972393 10.1016/j.apjtm.2016.01.035

[39] IDT. *OligoAnalyzer Program*, <https://www.idtdna.com/Scitools> (May 2023).

