# Supplementary material for: Detection and differentiation of low virulence and virulent Orthoavulavirus javaense using a molecular beacon with RT-LAMP
Source: Sci Rep. 2024 Aug 5;14:18047. doi: 10.1038/s41598-024-68816-7 (PMC11300668; doi:10.1038/s41598-024-68816-7)
Supplement: Supplementary file 1 — Supplementary Information. [file 41598_2024_68816_MOESM1_ESM.pdf]

Supplementary Table 1. Primer and probe sequences.

Name	Use	Sequence 5'-3'
4331F	Cloning	GAGGTTACCTCYACYAAGCTRGAGA
6344Rn	Cloning	ACCGTTCTACCCGTRTRTYGY
K13/4237F	Cloning	ACTCTGGAGTCAAACCGC
DR4297F	Cloning	GACTGCTGACCATGAGGTTAC
DR5100R	Cloning	CGTGCTGTATTGTTAAGCTGC
NEB Forward	Cloning	ACCTGCCAACCAAAGCGAGAAC
5755R	Cloning	TGCGATATGATWCCCGGRG
M+4100	qRT-PCR	AGTGATGTGCTCGGACCTTC
M-4220	qRT-PCR	CCTGAGGAGAGGCATTTGCTA
M+4169	qRT-PCR	[FAM]TTCTCTAGCAGTGGGACAGCCTGC[TAMRA]
F+4829	qRT-PCR	GGTGAGTCTATCCGGARGATACAAG
F-4939	qRT-PCR	AGCTGTTGCAACCCCAAG
F+4894	qRT-PCR	[FAM]AAGCGTTTCTGTCTCCTTCCTCCA[TAMRA]
F3-F-G3	MB-RT-LAMP	GAYAARGAGGCRTGTGC
B3-F-G3	MB-RT-LAMP	GCTGCTGTTATCTGTGC
FIP-F-G3	MB-RT-LAMP	CCTRCGGATRGAATCRCCAATTTTRGAGGCATACAACAGAACAY
BIP-F-G3	MB-RT-LAMP	AAGRGTCTGTGWCYACATCTGGTTTTAACCCCAAGAGCTACACY
B-Loop-F-G3	MB-RT-LAMP	AGGAGACARAAACGCTTTRTAGGTGC
F-Loop-F-G3	MB-RT-LAMP	GGRGTGAGYAAAGTRGTCA
V/M-F-G3	MB-RT-LAMP	[HEX]CGCACCAGGAGACARAAACGCTTTRTAGGTGCG[BHQ1]

Supplementary Table 1. Primer and probe sequences. Table shows primers used in this study. Non-standard genetic bases used include W = A or T, Y = C or T, R = A or G. FAM = 5' 6-fluorescein, TAMRA = 5-Carboxytetramethylrhodamine, HEX = Hexachloroflourescein, BHQ1 = Black hole quencher-1. F3 = Outer forward primer, FIP = Forward Inner Primer, BIP = Backward Inner Primer, B3 = Outer backward primer. FIP and BIP are composed of two binding sequences connected by a 4-nucleotide thymine linker (underlined).

Supplementary Table 2. Isolates used for MB-RT-LAMP primer design and assay.

Accession	Isolate Name	Pathotype	Genotype	ICPI
KC808494	Chachalaca/Mex(Chiapas)/668-ZM08/2009	Lento	I	0.00
MZ101338	Tadorna/Ukraine/Askania-Nova/3-20-11/2010	Lento	I	ND
KX352836	Teal/Novosibirsk region/320/2010	Lento	I	0.05
MK006018	Avian/USA/FL/475985/606/2007	Lento	I	0.04-0.09
AF077761	Chicken/USA/Lasota/1946	Lento	II	<0.4
KX822746*	TK80136	Lento	II	ND
MH996904	Pigeon/Bulgaria/NovoSelo/1161/1995	Velo	III	ND
EF201805	Karachi/NDV/Mukteshwar	Meso	III	1.4
MH996897	Chicken/Nigeria/Kano/N52/899/1973	Velo	IV	ND
EU293914*	Chicken/Haskovo 1968 Bulgaria	Velo	IV	ND
JN872181	Chicken/Honduras/44813/2000	Velo	V	ND
KU710273	Chicken/Bulgaria/Kardam/2008	Velo	V	1.86
MK583011	Chicken/Tanzania/Mbeya/MT15/2012	Velo	V	1.88
MK006006	Chicken/Mexico/NC/23/11/686/2011	Velo	V	1.89
KF767466	Belize (Spanish Lookout)/4224-3/2008	Velo	V	1.75
KP780873	Rock Pigeon/USA/MD/ND0007187	Velo	VI	0.66
KU059752	Dove/USA/TX/TX3988	Velo	VI	1.15
KX236101	Pigeon/Pakistan/Lahore/25A/2015	Velo	VI	1.46
AF431744	Goose/China/ZJ1/2000	Velo	VII	1.86
GU332646	Duck/Vietnam/Long Bien/78/2002	Velo	VII	1.88
MK673139*	Chicken/Egypt/136/2012	Velo	VII	1.88
MH001441	Chicken/Egypt/Qena/104/2012	Velo	VII	1.89
KF792021	Chicken/BT-Israel/2013/120	Velo	VII	ND
KY076030	Chicken/Pakistan/Sheikhupura/12A/994/2015	Velo	VII	1.89
MH392220	Poultry/China/04-23/C12/647/2004	Meso	IX	ND
GQ288391	Mottled duck/US(TX)/01-130/2001	Lento	X	0.04
MK006019	Turkey/USA/MN/P/TY/77/08/605/2008	Lento	X	0.00
MK633951	Chicken/Tanzania-Tanga/N1/2012	Velo	XIII	1.95
MK673140	Chicken/Tanzania/Tanga/N38/1317/2012	Velo	XIII	1.88
MH392222	Chicken/Pakistan/SPVC/Karachi/27/558/2007	Velo	XIII	1.64
KC568205	NG-705/KD.TW.7C	Velo	XIV	ND
MH996946	Chicken/Nigeria/KT/JBY/09T/N40/733/2009	Velo	XIV	ND
JX119193	Chicken/Dominican Republic (JuanLopez) /499-31/2008	Velo	XVI	1.88
MH996929	Duck/Nigeria/KG/LOM/11-16/N11/695/2009	Velo	XVII	1.89
KF442614	Nigeria/228-7/2006	Velo	XVII	1.9
MH392227	Chicken/Nigeria/OOT/4/1/N69/914/2009	Velo	XVIII	ND
MK673141	DCCO/USA/A00874288/650/2010	Velo	XIX	1.55
MK673141*	DCCO/USA/A00841380	Velo	XIX	1.39
GQ288387	Cormorant/US(MN)/92-40140/1992	Velo	XIX	ND
KY042125	Chicken/Bulgaria/Dolno Linevo/1160/1992	Velo	XX	ND
KY042127	Pigeon/Ukraine/Kharkiv/23-01/967/2013	Velo	XXI	1.7

Supplementary Table 2. Isolates used for MB-RT-LAMP primer design and assay. Table shows sequences used for MB-RT-LAMP primer and beacon design. NCBI accession numbers are indicated, those with * indicate sequences that are not submitted to GenBank and the accession number included is the most identical. Lento = Lentogenic, Meso = Mesogenic and Velo = Velogenic. ICPI = Intra-cerebral pathogenicity index. Only available ICPI values are included. ND = Not determined. Rows with gray shading are sequences chosen for IVT RNA.

Supplementary Table 3. Isolates used for MB-RT-LAMP assay and qRT-PCR comparison.

Accession	Isolate Name	Genotype	Patho-type	# Mismatches	ICPI
MG869263	NVNvacI2/Vietnam/2016	I	Lento	6	ND
AF217084	Chicken/Australia/Queensland/V4/1966	I	Lento	7	0.21
OP169005	Chicken/Georgia/2989/2021	I	Lento	5	0.11
EF564817	Ruddy Turnstone/DE/USA/492_/2002	I	Lento	8	0.00
AY562991	Chicken/Northern Ireland/Ulster/67	I	Lento	6	ND
EF564821	Mallard/US(MD)/04-204/2004	I	Lento	6	0.29
EF564816	Red Knot/US(NJ)/A101-1383/2001	I	Lento	8	0.08
EU289028	Turkey/USA/VG/GA/1989	II	Lento	6	ND
AF077761	Chicken/USA/Lasota/1946	II	Lento	6	<0.40
JN872151	Chicken/US/B1/48	II	Lento	6	0.04
FJ705464	Mallard/US(OH)/04-411/2004	X	Lento	8	0.40
FJ705467	Mallard/US(MN)/MN00-32/2000	X	Lento	8	0.05
FJ705469	Mallard/US(MN)/MN00-39/2000	X	Lento	8	0.18
GQ288378	Northern Pintail/US(OH)/87-486/1987	X	Lento	7	0.10
FJ705466	Mallard/US(MN)/99-376/1999	X	Lento	8	0.00
GU332645	Chicken/Australia/9809-19-1107/1998	I	Velo	4	1.88
X04719	Chicken/USA/Beaudette C/1945	II	Velo	1	1.75
MH996898	Chicken/Nigeria/Plateau/N53/900/1973	IV	Velo	1	ND
AY288999	Chicken/Mexico/37821/96	V	Velo	0	ND
MK040374	Chicken/California/B1800012/2018	V	Velo	0	ND
JN872192	Chicken/California/211472-4/2002	V	Velo	0	ND
MK583011	Chicken/Tanzania/Mbeya/MT15/2012	V	Velo	0	1.88
KP780870	Rock Pigeon/USA/MD/ND0002270/2013	VI	Velo	2	0.73
AY288996	Pigeon/Italy/1166/00	VI	Velo	2	0.80
GQ429293	Dove/Italy/2736/00	VI	Velo	2	1.25
JQ697739	Malaysia/1041-632/2008	VII	Velo	0	ND
OR230611 *	Chicken/Tanzania/Dar es Salaam/1996b/B03CL/2018 (swab)	VII	Velo	2	1.70
OR230611 *	Chicken/Tanzania/Dar es Salaam/1997b/B06CL/2018 (swab)	VII	Velo	2	1.71
KX791184*	Pk/O3/2018	VII	Velo	0	ND
KU295454	Chicken/Ukraine/Lyubotyn/961/2003	VII	Velo	0	ND
KU295453	Chicken/Ukraine/Kharkiv/66/960/2007	VII	Velo	0	ND
KU295455	Chicken/Ukraine/Bashtanivske/20-02/962/2013	VII	Velo	1	ND
KF767104	Cockatoo/Indonesia/1988/87-36724-524	VII	Velo	0	ND
AY562985	Cockatoo/Indonesia/14698/90	VII	Velo	0	ND
MK005979	Chicken/Egypt/Sohag/21/1023/2011	VII	Velo	1	ND
JN800306	Chicken/Peru/1918-03/603/2008	XII	Velo	1	1.78
MK673140	Chicken/Tanzania/Tanga/N38/1317/2012	XIII	Velo	1	1.88
KC568206	NG-706/JG.KZ.14T	XIV	Velo	4	ND
FJ772463	Chicken/2415/580/Burkina Faso/2008	XVII	Velo	0	1.69
KU058680	Duck/Nigeria/903/KUDU-113/1992	XVII	Velo	0	1.88

MH996936	Guinea fowl/Nigeria/YB/GSHI/9-10C/N9/694/2009	XVII	Velo	2	ND
MH996925	Chicken/Nigeria/BA/TFB/14C/N38/711/2009	XVII	Velo	2	ND
MH996932	Vulture/Nigeria/PL038-XVII/N47/895/2002-2003	XVII/II/OAVJ3	Velo	0	ND
MH392227	Chicken/Nigeria/OOT/4/1/N69/914/2009	XVIII	Velo	0	ND
MH392216	Cormorant/USA/MN/92-40140/250/1992	XIX	Velo	1	1.39
KY042142	Quail/South Korea/88-M/514/1988	XX	Velo	1	ND
KY042143	Chicken/South Korea/93-58GG/548/1993	XX	Velo	1	ND
KJ914671	Pigeon/Ukraine/Dnipropetrovsk/1-18-11/2011	XXI	Velo	2	1.15
KJ914672	Pigeon/Ukraine/Ukromne/3-26-11/2011	XXI	Velo	2	1.36
KY042131	Pigeon/Egypt/Qena/56/1107/2015	XXI	Velo	2	1.88
KY042129	Pigeon/Egypt/Giza/11/1088/2015	XXI	Velo	2	1.31
KY042128	Pigeon/Ukraine/Doneck/3/968/2007	XXI/II	Velo	2	1.48
N/A	88-20016-Phillipines	Unk	Susp. Velo	Unk	ND
N/A	88-25393 Mexico	Unk	Susp. Velo	Unk	ND
N/A	91-11408-Singapore	Unk	Susp. Velo	Unk	ND
N/A	93-28710-Tanzania/Belgium/China	Unk	Susp. Velo	Unk	ND

Supplementary Table 3. Isolates used for MB-RT-LAMP assay and qRT-PCR comparison. Table shows isolates used for MB-RT-LAMP and qRT-PCR comparison. NCBI accession numbers are indicated, those with * indicate sequences that are not submitted to GenBank and the accession number included is the most identical. Lento = Lentogenic, Meso = Mesogenic and Velo = Velogenic. Roman numerals indicate genotype. Mismatches are calculated by comparison to molecular beacon sequence. ICPI = Intra-cerebral pathogenicity index. Only available ICPI values are included. ND = Not determined.

Supplementary Table 4. Detergent combinations tested and their impact on MB-RT-LAMP.

Detergent	Final Concentration	Result
NP-40	0.05%	Nonspecific amplification
NP-40	0.1%	Nonspecific amplification
NP-40	0.5%	Nonspecific amplification
NP-40/SDS	0.05% / 0.01%	Nonspecific amplification
NP-40/SDS	0.1% / 0.01%	Nonspecific amplification
OBG	0.04%	Reduced beacon differentiation and no viral lysis
SDS	0.01%	Reduced beacon differentiation
SDS/BSA	0.1% / 5μg	LAMP inhibition
SDS/TnBP	0.01% / 0.1%	Inconsistent viral lysis
SDS/Tween-80	0.01% / 0.1%	No viral lysis
SDS/TnBP/Tween-80	0.01% / 0.1% / 0.1%	Consistent viral lysis and good beacon differentiation of isolates
TnBP	0.1%	No amplification impairment
TnBP/Triton X-100	0.1% / 0.5%	Changed mastermix color
TnBP/Tween-80	0.03% / 0.1%	No amplification impairment
TnBP/Tween-80	0.1% / 0.1%	Slightly improved beacon differentiation
TnBP/Tween-80	1% / 1%	Changed mastermix color
Triton X-100	0.1%	Nonspecific amplification
Triton X-100	0.2%	Nonspecific amplification
Triton X-100	0.5%	Changed mastermix color and nonspecific amplification
Tween-20	0.5%	Changed mastermix color
Tween-80	0.1%	No amplification impairment

Supplementary Table 4. Detergent combinations tested and their impact on MB-RT-LAMP. Table shows the different detergents tested individually or in various combinations, listed alphabetically, and their impact on the MB-RT-LAMP reaction. The final concentration of each detergent as it was used in the MB-RT-LAMP reaction is shown, but the concentration of the detergent if it was tested as a lysis buffer was 10-fold greater (i.e., SDS was used as a lysis buffer at 0.1% for a final concentration of 0.01% in the MB-RT-LAMP as the input used was only 10% of the final reaction volume). Grey shaded row indicates detergent combination used for rapid lysis workflow. NP-40 = Nonidet P40. OBG = Octyl-betaglucoside. TnBP = Tri(n-butyl) phosphate. SDS = Sodium dodecyl sulfate.

Supplementary Figure 1: Optimization of MB-RT-LAMP assay primer concentration and chemical additions.

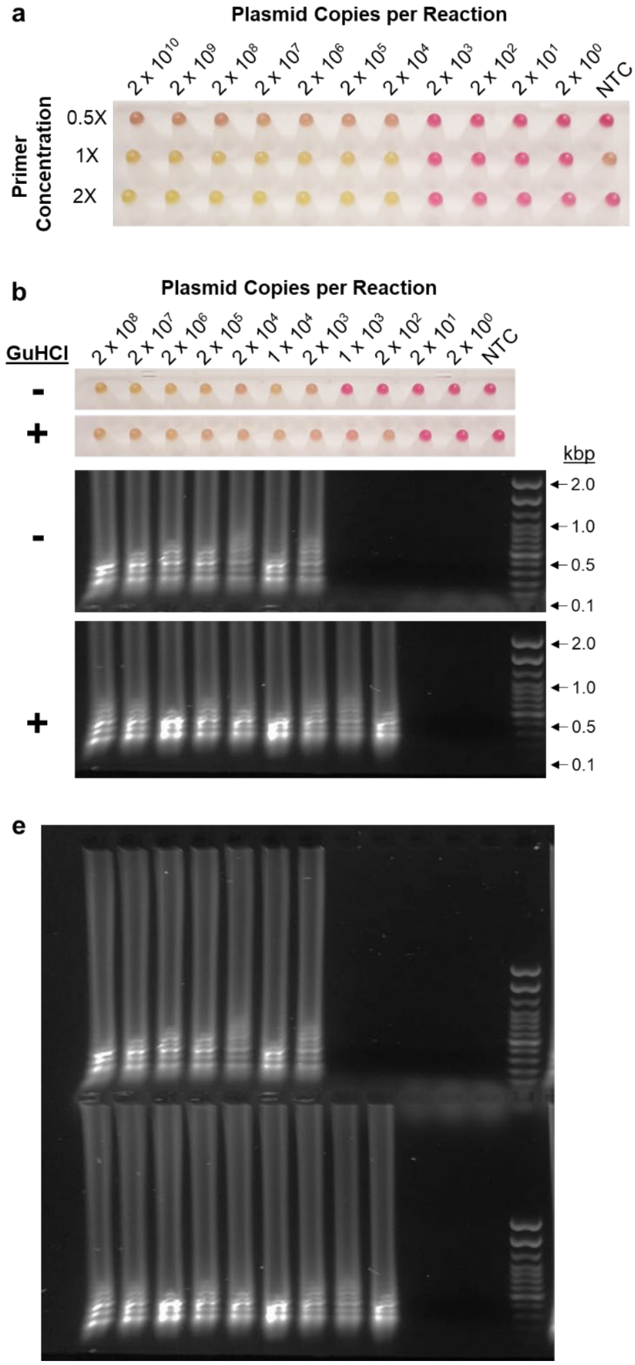

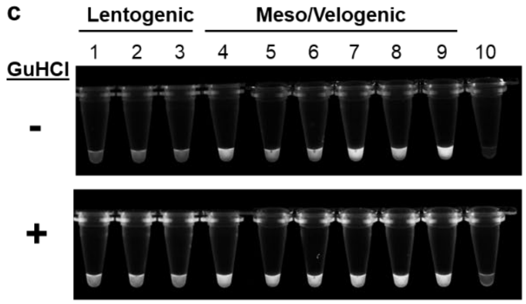

Tube	Isolate	Pathotype
1	Chachalaca/Mex(Chiapas)/668-ZM08/2009	Lento
2	Chicken/USA/Lasota/1946	Lento
3	TK80136	Lento
4	Chicken/DominicanRepublic (JuanLopez)/499-31/2008	Velo
5	Karachi/NDV/Mukteshwar	Meso
6	Chicken/Nigeria/Kano/N52/899/1973	Velo
7	Poultry/China/04-23/C12/647/2004	Meso
8	Chicken/Haskovo 1968 Bulgaria	Velo
9	Chicken/Tanzania/Mbeya/MT15/2012	Velo
10	No Template Control	N/A

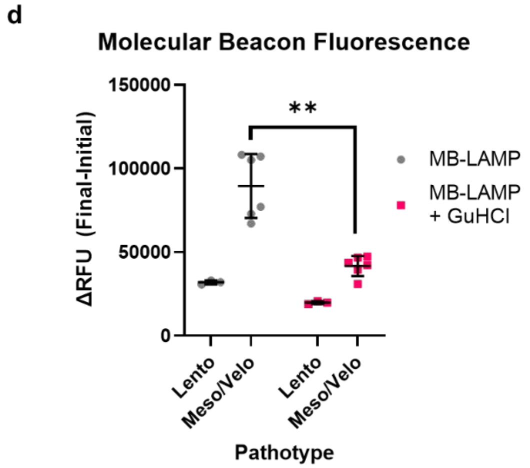

Supplementary Figure 1. Optimization of MB-RT-LAMP assay primer concentration and chemical additions. a) Optimizing primer concentration for colorimetric LAMP. LAMP reactions containing LaSota fusion plasmid at 2 to 2 x 10^{10} copies were run with 0.5X, 1X and 2X concentration of LAMP primer mix. Yellow color indicates amplification, pink color indicates no

amplification. NTC = No template control. **b)** LAMP reactions were run with 2 to 2 x 10^8 copies of LaSota fusion plasmid template in the absence or presence of 40mM guanidine hydrochloride (GuHCl). Top panel shows colorimetric endpoint where yellow color indicates amplification and pink color indicates no amplification. NTC = No template control. Bottom panel shows gel electrophoresis of RT-LAMP products. Molecular marker sizes are indicated in kilobase pairs (kbp) to the right of the 100bp molecular marker. **c)** Fluorescence endpoint image of fusion gene plasmids of different pathotypes tested using MB-RT-LAMP in the absence or presence of 40mM GuHCl. Table below image panels describes isolates chosen and their pathotype. **d)** Quantification of molecular beacon fluorescence from samples in panel c. Plot shows the net difference in molecular beacon fluorescence binding (ΔRFU Final – Initial) for each lentogenic and mesogenic/velogenic fusion template. Error bars represent mean ± standard deviation for each group. Statistical difference of the mean ΔRFU between the mesogenic/velogenic groups in the absence or presence of 40mM GuHCl was calculated using a two-tailed unpaired student's t-test with Welch's correction ($p=0.0011$). **e)** Complete image of electrophoresis gel for cropped gel image in panel b.

Supplementary Figure 2. Molecular beacon differentiation of lentogenic and mesogenic/velogenic isolates is improved after sample cooling to room temperature before measuring fluorescence.

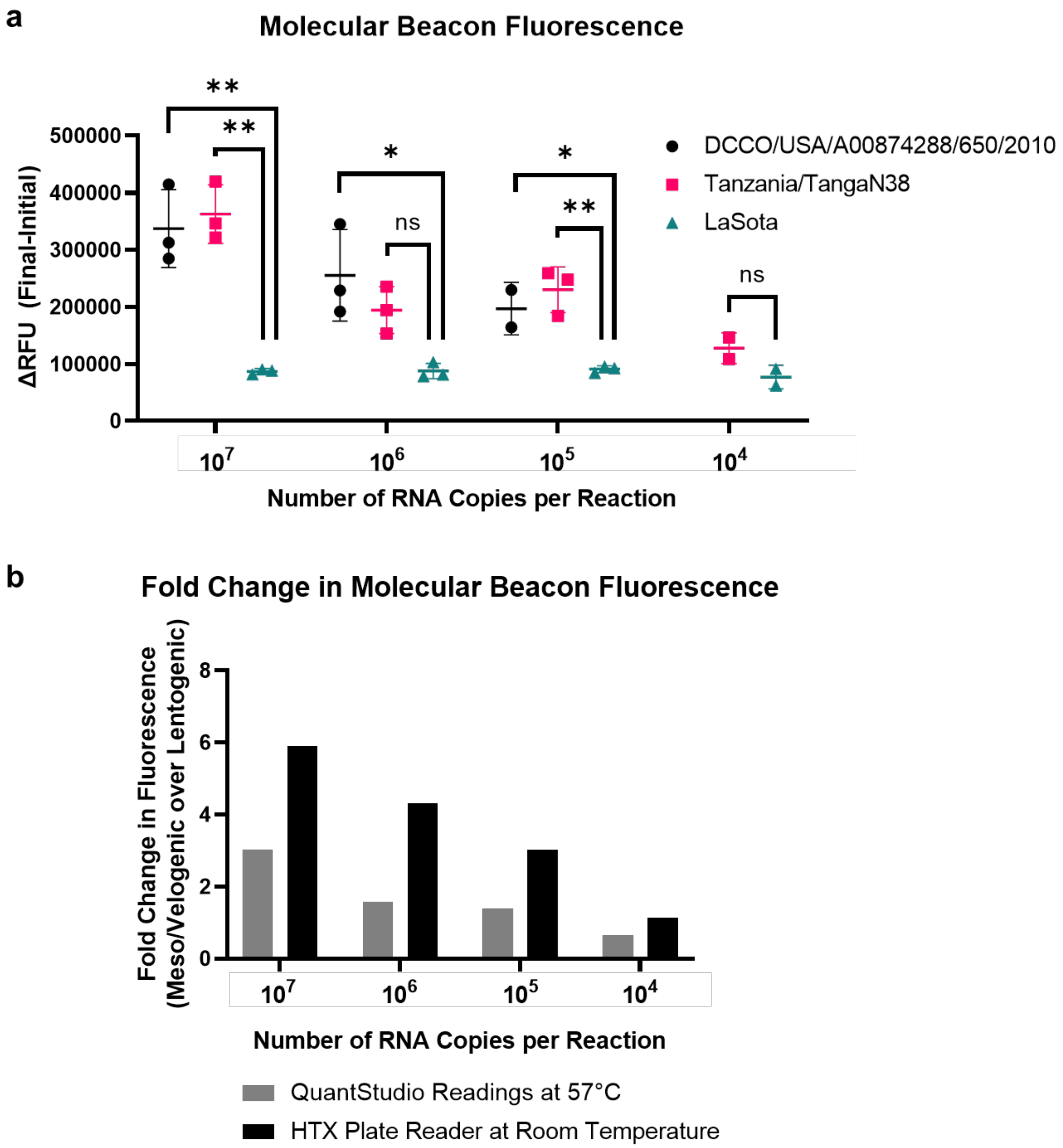

Supplementary Figure 2. Molecular beacon differentiation of lentogenic and mesogenic/velogenic isolates is improved after sample cooling to room temperature before measuring fluorescence. a) Quantification of molecular beacon fluorescence at the end of incubation at 57°C. Samples correspond to those shown in Figure 3. Fluorescence difference is less significant than samples cooled to room temperature before reading fluorescence on a plate reader (Figure 3b). **b)** Fold change in fluorescence of mesogenic/velogenic isolates over

lentogenic isolates when calculated post-incubation at 57°C or after cooling to room temperature and measuring on a plate reader. A greater fold change in fluorescence between velogenic and lentogenic templates is seen in reactions that have been cooled to room temperature then measured.

Supplementary Figure 3. Evaluation of the accuracy of the MB-RT-LAMP assay compared to qRT-PCRs.

a

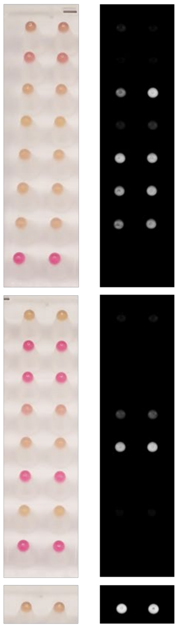

Isolate Name:	LAMP	Beacon	Beacon Correct?	Fusion rRT-PCR	Fusion rRT-PCR Correct?	Matrix rRT-PCR
NVNvacl2/Vietnam/2016			Y	-	Y	+
Chicken/Australia/Queensland/V4/1966			Y	-	Y	+
Chicken/Georgia/2989/2021			N	-	Y	+
Turkey/USA/VG/GA/1989			Y	-	Y	+
Mallard/US(OH)/04-411/2004			N	-	Y	+
Mallard/US(MN)/MN00-32/2000			N	-	Y	+
Mallard/US(MN)/MN00-39/2000			N	-	Y	+
Ruddy Turnstone/DE/USA/492_/2002			N/A	-	Y	+
Chicken/US/B1/48			Y	-	Y	+
Chicken/Northern Ireland/Ulster/67			N/A	-	Y	+
Red Knot/US(NJ)/A101-1383/2001			N/A	-	Y	+
Northern Pintail/US(OH)/87-486/1987			N	-	Y	+
Mallard/US(MN)/99-376/1999			N	-	Y	+
Mallard/US(MD)/04-204/2004			N/A	-	Y	+
Chicken/USA/Lasota/1946			Y	-	Y	+
No Template Control			Y	-	Y	-
Velo Positive Control: ZJ1 IVT RNA			Y	+	Y	N/A
	**11/15 73.3%**		**5/11 45.5%**		**15/15 100%**	**15/15 100%**

Supplementary Figure 3. Evaluation of the accuracy of the MB-RT-LAMP assay compared to qRT-PCRs. Total RNA was extracted from a variety of virus stocks representing diverse isolates from 15 different genotypes of both lentogenic and velogenic pathotypes. Three assays were completed in parallel, using technical duplicates for each sample in each assay: 1) MB-RT-LAMP, 2) OAVJ F gene qRT-PCR, and 3) OAVJ M gene qRT-PCR. **a)** Total RNA was tested from 15 lentogenic isolates. Left panel shows the colorimetric endpoint of the assay followed by the fluorescent endpoint of the assay. Tabulated results on the right show the accuracy of the molecular beacon fluorescence, and results of the OAVJ F gene and M gene qRT-PCR results. **b)** Total RNA was tested from 41 velogenic isolates. Left panel shows the colorimetric endpoint of the assay followed by the fluorescent endpoint of the assay. Tabulated results on the right show the accuracy of the molecular beacon fluorescence, and results of the OAVJ F gene and M gene qRT-PCR results. **c)** Graph shows the mean molecular beacon fluorescence from technical duplicates of all samples amplified by MB-RT-LAMP assay. Samples are separated by correct differentiation by molecular beacon (N = 36) or incorrect differentiation (N = 9).

b

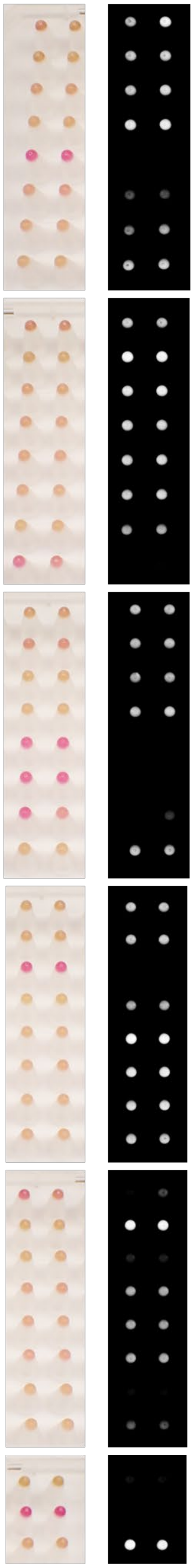

**Isolate Name**:	**LAMP**	**Beacon**	**Beacon Correct?**	**Fusion rRT-PCR**	**Fusion rRT-PCR Correct?**	**Matrix rRT-PCR**
Chicken/Tanzania/Mbeya/MT15/2012			Y	+	Y	+
Chicken/Tanzania/Tanga/N38/1317/2012			Y	+	Y	-
Chicken/Peru/1918-03/603/2008			Y	+	Y	+
Chicken/Nigeria/OOT/4/1/N69/914/2009			Y	+	Y	-
Pigeon/Egypt/Qena/56/1107/2015			N/A	-	N	-
Chicken/Egypt/Sohag/21/1023/2011			Y	-	N	+
Cockatoo/Indonesia/14698/90			Y	+	Y	+
Cockatoo/Indonesia/1988/87-36724-524			Y	+	Y	+
88-25393 Mexico			Y	+	Y	+
88-20016-Phillipines			Y	+	Y	-
91-11408-Singapore			Y	+	Y	+
Chicken/California/B1800012/2018			Y	+	Y	+
Chicken/California/211472-4/2002			Y	+	Y	+
93-28710-Tanzania/Belgium/China			Y	+	Y	+
Chicken/Nigeria/Plateau/N53/900/1973			Y	+	Y	+
Chicken/Australia/9809-19-1107/1998			N/A	-	N	+
Chicken/USA/Beaudette C/1945			Y	+	Y	+
Chicken/Mexico/37821/96			Y	+	Y	+
Pigeon/Italy/1166/00			Y	+	Y	+
Dove/Italy/2736/00			Y	-	N	+
Chicken/Tanzania/Dar es Salaam/1996b/B03CL/2018 (swab)			N/A	-	N	-
Chicken/Tanzania/Dar es Salaam/1997b/B06CL/2018 (swab)			N/A	-	N	-
Pk/O3/2018			N/A	+	Y	+
Cormorant/USA/MN/92-40140/250/1992			Y	+	Y	+
Quail/South Korea/88-M/514/1988			Y	+	Y	+
Chicken/South Korea/93-58GG/548/1993			Y	+	Y	+
Pigeon/Egypt/Giza/11/1088/2015			N/A	-	N	-
Rock Pigeon/USA/MD/ND0002270/2013			Y	-	N	+
Malaysia/1041-632/2008			Y	+	Y	+
Chicken/Ukraine/Lyubotyn/961/2003			Y	+	Y	+
Chicken/Ukraine/Kharkiv/66/960/2007			Y	+	Y	+
Chicken/Ukraine/Bashtanivske/20-02/962/2013			Y	+	Y	+
NG-706/JG.KZ.14T			N/A	-	N	+
Duck/Nigeria/903/KUDU-113/1992			Y	+	Y	+
Vulture/Nigeria/PL038-XVII/N47/895/2002-2003			N	+	Y	+
Guinea fowl/Nigeria/YB/GSHI/9-10C/N9/694/2009			Y	+	Y	+
Chicken/Nigeria/BA/TFB/14C/N38/711/2009			Y	+	Y	+
Chicken/2415/580/Burkina Faso/2008			Y	+	Y	-
Pigeon/Ukraine/Dnipropetrovsk/1-18-11/2011			N	-	N	+
Pigeon/Ukraine/Ukromne/3-26-11/2011			Y	+	Y	+
Pigeon/Ukraine/Doneck/3/968/2007			N	-	N	+
No Template Control			Y	-	Y	-
Velo Positive Control: ZJ1 IVT RNA			Y	+	Y	N/A
	**34/41 82.9%**		**31/34 91.2%**		**30/33 90.9%**	**33/41 80.5%**

c

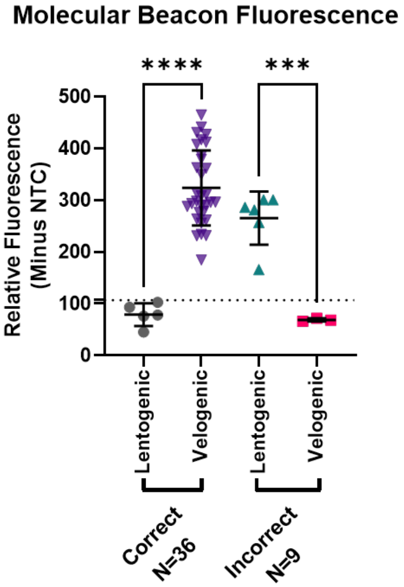

Molecular Beacon Fluorescence
****
***
Relative Fluorescence (Minus NTC)
500
400
300
200
100
0
Lentogenic
Velogenic
Lentogenic
Velogenic
Correct N=36
Incorrect N=9

Supplementary Figure 4. The rapid lysis method is effective for detection of OAVJ RNA from a variety of egg passaged lentogenic isolates and is not inhibited by oropharyngeal swab material.

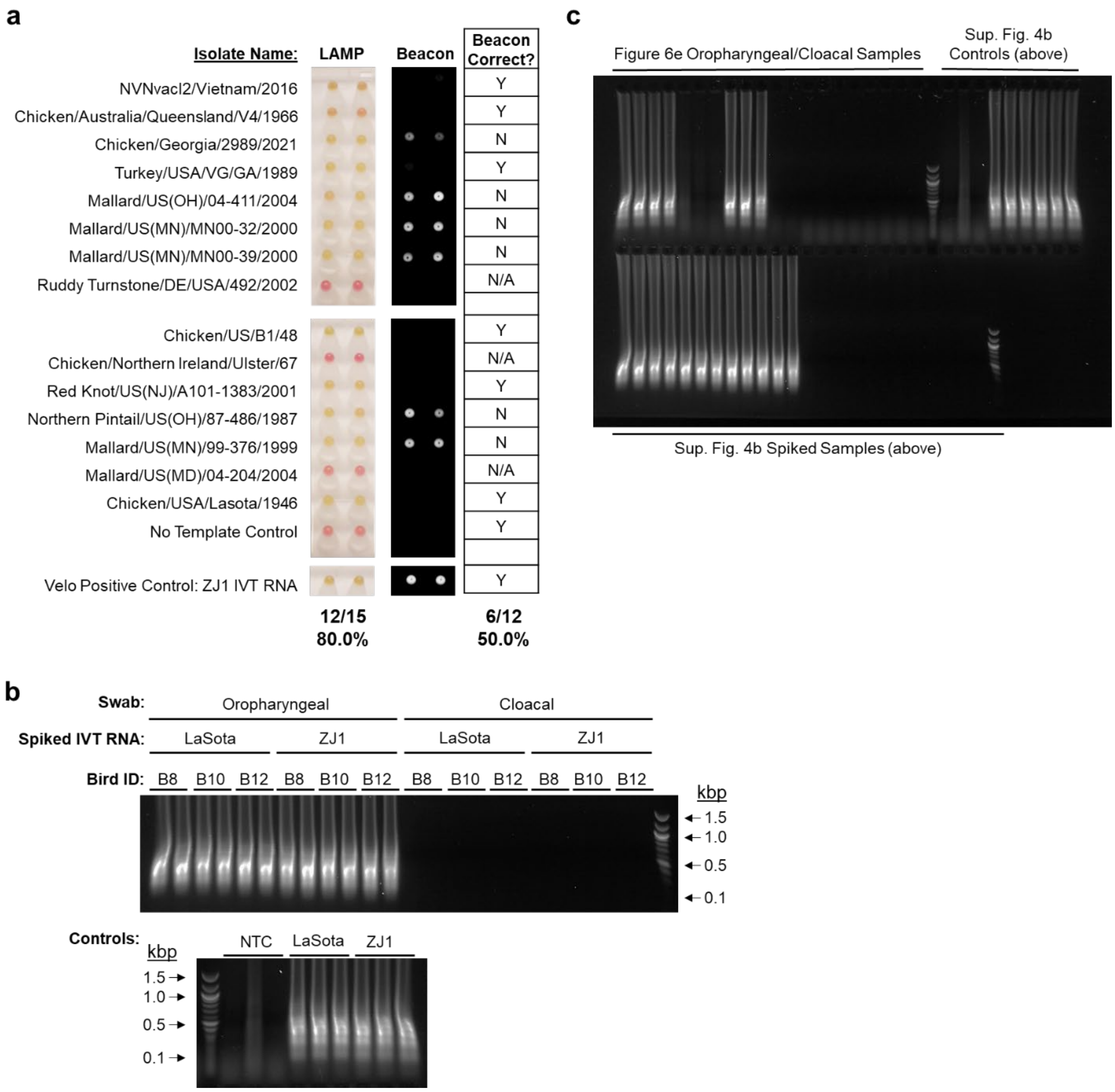

Supplementary Figure 4. The rapid lysis method is effective for detection of OAVJ RNA from a variety of egg passaged lentogenic isolates and is not inhibited by oropharyngeal swab material. a) Swabs from 15 different egg passaged lentogenic isolates were tested using the rapid lysis to MB-RT-LAMP workflow. Left panel shows the colorimetric endpoint of the assay followed by the fluorescent endpoint of the assay. Tabulated results on the right show the accuracy of the molecular beacon fluorescence. The rapid lysis method enabled detection of 12/15 isolates (80%). **b)** Gel electrophoresis of MB-RT-LAMP reactions using LaSota or ZJ1 F gene IVT RNA spiked into the SDS rapid lysis tube, then treated with oropharyngeal or cloacal swab material from three different specific pathogen free chicks swabbed at 7 days post-hatch.

Cloacal swab material inhibited amplification of the MB-RT-LAMP reaction, as shown by no ladder banding pattern typical of LAMP reaction products. The typical ladder banding pattern is seen in the oropharyngeal swab reactions and controls, suggesting that oropharyngeal swab material does not inhibit the MB-RT-LAMP reaction. **c)** Complete image of electrophoresis gel for cropped gel image in panel b. Samples on the top left correspond to B4, B6, B8, B10, and B11, oropharyngeal then cloacal swab technical duplicates from experiment shown in Figure 6e. Samples on the top right are the controls as shown in panel b, and samples on the bottom are the IVT RNA spiked samples shown in panel b.

Supplementary Figure 5. Predicted stability of beacon binding to fusion cleavage sites.

Strain	# Matching bp	# Mis-matches	ΔG (kcal/mol)	Beacon signal (Y/N)	Phe 117 (Y/N)
Chicken/Tanzania/Mbeya/MT15/2012	27	0	-52.25	Y	Y
Turkey/USA/MN/P/TY/77/08/605/2008	14	4	-27.17	Y	Y
Avian/USA/FL/475985/606/2007	12	5	-22.07	Y	Y
Chicken/Georgia/2989/2021	11	5	-20.73	Y	Y
Mottled duck/US(TX)/01-130/2001	10	7	-16.87	Y	N
Northern Pintail/US(OH)/87-486/1987	10	7	-16.87	Y	N
Mallard/US(MN)/MN00-32/2000	10	8	-16.87	Y	N
Chicken/USA/Lasota/1946	8	6	-12.88	N	N

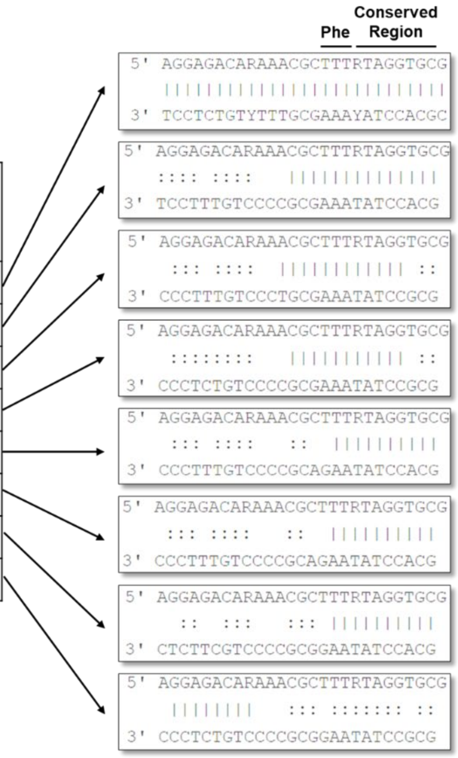

Supplementary Figure 5. Predicted stability of beacon binding to fusion cleavage sites. The heterodimer stability between the molecular beacon and various fusion cleavage sites was calculated using the IDT OligoAnalyzer tool. Figure shows the fusion cleavage sites used, the number of base pairs that are predicted to bind to the beacon, the total number of mismatches between the fusion cleavage site sequence and the beacon, the predicted ΔG (Kcal/mole) of binding, whether the beacon gave positive signal when tested, and whether the fusion cleavage site contained a Phe residue at position 117 (left table). Images on the right show the predicted heterodimeric duplex of the molecular beacon (top sequence, 5’ to 3’) with the reverse complement of each fusion cleavage site (bottom sequence, 3’ to 5’).

Supplementary Figure 6: Complete image of electrophoresis gel for cropped gel image in Figure 2a.

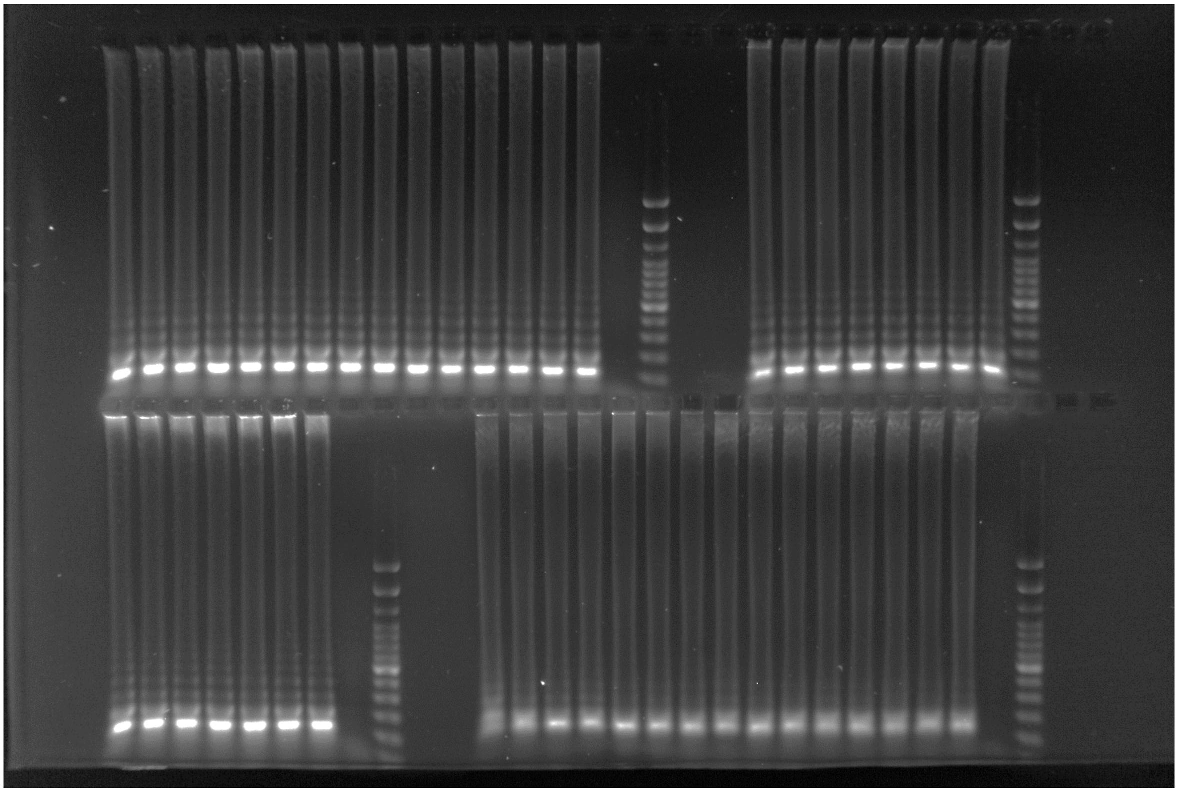

Supplementary Figure 6: Complete image of electrophoresis gel for cropped gel image in Figure 2a. The same 15 samples plus no template control listed in Figure 2a are shown from three experiments conducted at different temperatures. Samples on the top left were incubated at 53°C, samples on the top right and bottom left were incubated at 55°C, and samples on the bottom right were incubated at 57°C. The samples on the bottom right correspond to the cropped lanes shown in Figure 2a. There are two empty lanes to the right of each 100bp molecular ladder
